# Distinct Temporal Succession of Bacterial Communities in Early Marine Biofilms in a Portuguese Atlantic Port

**DOI:** 10.3389/fmicb.2020.01938

**Published:** 2020-08-11

**Authors:** Jorge T. Antunes, António G. G. Sousa, Joana Azevedo, Adriana Rego, Pedro N. Leão, Vitor Vasconcelos

**Affiliations:** ^1^Centro Interdisciplinar de Investigação Marinha e Ambiental, Universidade do Porto, Matosinhos, Portugal; ^2^Departamento de Biologia, Faculdade de Ciências, Universidade do Porto, Porto, Portugal; ^3^Institute of Biomedical Sciences Abel Salazar, University of Porto, Porto, Portugal

**Keywords:** marine biofilms, marine corrosion, marine fouling, pioneer bacteria, steel surfaces, temporal succession

## Abstract

Marine biofilms are known to influence the corrosion of metal surfaces in the marine environment. Despite some recent research, the succession of bacterial communities colonizing artificial surfaces remains uncharacterized in some temporal settings. More specifically, it is not fully known if bacterial colonizers of artificial surfaces are similar or distinct in the different seasons of the year. In particular the study of early biofilms, in which the bacterial cells communities first adhere to artificial surfaces, are crucial for the development of the subsequent biofilm communities. In this work, we used amplicon-based NGS (next-generation sequencing) and universal 16S rRNA bacterial primers to characterize the early biofilm bacterial communities growing on 316 L stainless steel surfaces in a Northern Portugal port. Sampling spanned 30-day periods in two distinct seasons (spring and winter). Biofilm communities growing in steel surfaces covered with an anti-corrosion paint and planktonic communities from the same location were also characterized. Our results demonstrated that distinct temporal patterns were observed in the sampled seasons. Specifically, a significantly higher abundance of Gammaproteobacteria and Mollicutes was found on the first days of biofilm growth in spring (day 1 to day 4) and a higher abundance of Alphaproteobacteria during the same days of biofilm growth in winter. In the last sampled day (day 30), the spring biofilms significantly shifted toward a dominance of photoautotrophic groups (mostly diatoms) and were also colonized by some macrofouling communities, something not observed during the winter sampling. Our results revealed that bacterial composition in the biofilms was particularly affected by the sampled day of the specific season, more so than the overall effect of the season or overall sampling day of both seasons. Additionally, the application of a non-fouling-release anti-corrosion paint in the steel plates resulted in a significantly lower diversity compared with plates without paint, but this was only observed during spring. We suggest that temporal succession of marine biofilm communities should be taken in consideration for future antifouling/anti-biofilm applications.

## Introduction

Once a surface is submerged in seawater, it is quickly covered with a layer of adsorbed molecules, mostly proteins and glycoproteins, which form a conditioning film before the attachment of any microbial cells (Cooksey and Wigglesworth-Cooksey, 1995; Flemming and Wingender, 2010). Afterwards, there is the colonization of surface-associated aggregates of microbes (including bacteria, fungi and protists) which constitute marine biofilms (Dang and Lovell, 2016; Antunes et al., 2018). Although the primary marine biofilm is dominated by bacterial species, especially after immersion for a few days, a significant accumulation of biomass is also attributed to diatoms (Railkin, 2004; Hunsucker et al., 2014). Other groups of microorganisms like cyanobacteria, benthic dinoflagellates, fungi and protozoa follow the colonization after a few days, resulting in the formation of mature marine biofilms (Molino and Wetherbee, 2008). Mature biofilm are biofilms characterized by an organized structure surrounded by extracellular polymeric substances (EPS) and interspersed with fluid-filled channels (Wahl et al., 2012). The development of microbial communities in artificial surfaces may be due to surface properties that are expressed in the presence of the ubiquitous film of dissolved organic matter or to unique properties of the adsorbed film other than bulk chemical composition or previous attached communities (Ralston and Swain, 2014). There is also a dynamic equilibrium with this component of the substrate with the flocculation of marine snow and the water column. The build-up of a biofilm is a complex process in which the composition of whatever accumulates is based primarily upon the numbers of each kind of item available (Little et al., 1986; Clare et al., 1992). The microbial communities that initially attach to surfaces are believed to be the triggers of the marine biofouling processes (Salta et al., 2013) as marine biofilms may constitute substrates for the colonization and settlement of larvae and of spores of higher sessile organisms like macroinvertebrates (Dobretsov et al., 2009; Hadfield, 2011). However, the interaction between bacteria and macrofouling organisms is dynamic and varied, and certain types of microbial fouling may inhibit the settlement of macrofouling organism, while other macrofouling organisms are known to settle in surfaces after a few days without any interaction with biofilms (Maki et al., 1990; Dobretsov and Rittschof, 2020).

Biofilms have a significant role in the corrosion of steel surfaces immersed in seawater (Dang et al., 2011; de Carvalho, 2018), and are responsible for diminished hydrodynamic performance in ships (Schultz and Swain, 2000; Holm et al., 2004) and can also be vectors for the spread of pathogenic bacteria (Shikuma and Hadfield, 2010; Oberbeckmann et al., 2016; Flach et al., 2017; Sathe et al., 2017). Certain bacterial taxa are routinely described as early colonizers of metal surfaces and are present in low-water corrosion (ALWC) structures (McBeth et al., 2011; Jin et al., 2015), a problem with a significant economic impact (Hou et al., 2017). Despite several published studies in the last years, the characterization of early marine biofilm communities, the bacterial cell communities that first adhere to a surface, in different environmental settings remains incomplete (Salta et al., 2013; Lawes et al., 2016; de Carvalho, 2018). It is believed that biofilm and planktonic prokaryotic communities are distinct even from the first stages of biofilm formation. However, microbial biofilms in aquatic environments are very heterogeneous and dynamic and are influenced by the surrounding environmental parameters (such as nutrient status, temperature, hydrodynamics and water chemistry) as well by the physicochemical properties of solid surfaces and latitudinal gradient (Ward et al., 2003; Jones et al., 2007; Fuhrman et al., 2008; Milici et al., 2016), temperature, as well biogeochemical and physical interactions, seasonal and geographical variations (Kim et al., 2016). It is believed there is a complex relationship between the nature of the metal substratum, the organic conditioning film and the attachment and detachment of bacteria (Maki et al., 1990). Similarly, biofilm temporal succession may have formed a consistent time series, each being associated with a different bacterial community, but those patterns have not been thoroughly analyzed in some temporal settings (Stoodley et al., 2002; Briand et al., 2012, 2017). The impact of temporal variations in the evolution of these pioneer biofilm microbial communities may be important to determine the correct application timeframe and for the selection of anti-fouling or anti-corrosion paints (Antunes et al., 2018).

The ability to characterize biofilm community composition in environmental samples has increased substantially through the use of high-throughput next-generation gene sequencing (NGS)-based methodologies allowing for unprecedented analyses of microbiota (Besemer et al., 2012). Previous studies have characterized the bacterial biofilm communities growing on different artificial substrata or on different geographical locations (Briand et al., 2012; Dobretsov et al., 2013; Elifantz et al., 2013; Briand et al., 2017; Pollet et al., 2018). Overall, the current body of knowledge regarding bacterial communities of marine biofilms indicates that Proteobacteria, and in particular the Alphaproteobacteria class dominate the colonization of artificial surfaces (Dang and Lovell, 2000; Huggett et al., 2006; Jones et al., 2007; Chung et al., 2010). Roseobacter clade members (Alphaproteobacteria) seem to be the dominant group colonizing artificial surfaces immersed in temperate coastal waters in the Atlantic and the Pacific Ocean (Dang et al., 2008). Our main objective in this study was therefore to characterize the temporal succession of bacterial marine biofilm communities, over a 1-month period growing on steel surfaces both during winter and during spring. We also aimed at characterizing the biofilm communities growing in surfaces covered with a common a non-release anti-corrosion paint following a 30-day immersion period, which would allow to determine if the product would have an effect on the bacterial biofilm communities, particularly the ones believed to influence the corrosion of steel plates (Li et al., 2016, 2017).

## Materials and Methods

### Study Site and Sampling

The Port of Leixões, North of Portugal (Matosinhos) was selected for the field assays (41°10′39.32′′N, 8°42′8.78′′W) (Supplementary Figure S1) and is a multipurpose port being simultaneously a terminal for ocean-going passenger ships and for cargo ships. This port, located next to the River Leça mouth, was selected because submerged artificial surfaces in that location are known to be significantly covered with marine. A pier in the northwest part of the Leixões Cruise Terminal marina was selected as the study site (see Supplementary Figure S1). As substrate for biofilm growth, we employed stainless steel foil plates (10 × 10 × 0.2 cm) of the 316 L type, an austenitic chrome nickel steel with superior corrosion resistance typically used in surfaces exposed to chemical corrodents as well as submerged in marine environments. These plates have a chemical composition of a maximum 0.08% carbon, 2.00% manganese, 0.75% silicon, 0.045% phosphorous, 0.03% sulfur, 16–18% chromium, 2–3% molybdenum, and 10–14% nickel. Afterward, the plates were submerged vertically on a pier deck in the Leixões cruise terminal at a constant depth of approximately 1 m (due to the floating system of the platform) (see Supplementary Figure S1). Two sampling periods were conducted over two contrasting seasons of the year: during the spring of 2016 (24th of May–23rd of June 2016), and during the winter of 2017 (7th of February–8th of March 2017). Three panels per season were professionally covered with Intersheen 579 (International Paint Ltd, Gateshead, United Kingdom), by the Port Authority of Douro, Leixões and Viana do Castelo (APDL), and sampled on the day 30 of each season, to determine the effect of this product in the biofilm structure in the last day of sampling. The painted panels were professionally covered with Intersheen 579 (International Paint Ltd, Gateshead, United Kingdom) a non-biocidal inert modified acrylic finish which is not fouling released, offering corrosion protection of surfaces, following previous studies (Macleod et al., 2016). The anti-corrosive performance of this product is due to acrylic barrier protection, it is a non-ablative, standard surface product without additives of fouling-release (FR) properties. The plates steel surfaces were cleaned with high pressure freshwater by the APDL personnel to remove possible soluble contaminants and foreign matter and then the plates were air dried. The paint was afterward mixed thoroughly with a power agitator and applied using a brush and left to dry for 24 h following manufacturer’s instructions.

The plates were divided into groups of three plates sampled for each day of each sampling season, plus a triplicate of plates covered with anti-corrosion paint, making a total of 60 independent steel plates. The stainless-steel plates were attached by a kernmantle rope, supported by a rope which was attached to a submerged gray PVC tube vertically placed within a distance of 10 cm from each other (in order to avoid contact between plates), and positioned with a northeast/southwest orientation. Plates were placed with a Northeast orientation taking in consideration that the Northeast-facing side was more exposed to sunlight, while the opposite orientation was facing to a wall of the marina which was covering the sunlight (see Supplementary Figure S1). All plates were permanently immersed throughout the sampling campaign, and were only re-surfaced to conduct the sampling and were then vertically submerged with the same orientation. To conduct the sampling, an independent set of three non-coated steel plates were retrieved at days 1, 2, 4, 7, 10, 14, 21, 25, and 30 of immersion in each of the sampled seasons, in order to obtain triplicate samples for each of the sampled days. A further set of three coated steel plates were sampled on the last day of immersion of each season. The triplicates plates for each of the sampling condition were randomly selected in order to minimize the effect of the allocated position within each of the monitoring structure attached to the rope. After each time of immersion, the steel plates were rinsed with sterile seawater (0.2 μm pore sized filtered) to remove sediment and suspended cells which were not permanently attached to the surfaces (Chen et al., 2013). Afterward, the biofilms attached to the steel plates after washing/rinsing (see Supplementary Figure S1) were collected by scraping using a sterile razor (Briand et al., 2012; Camps et al., 2014) and stored into sterilized microcentrifuge tubes. Since the panels in the last days of immersion were fouled with macro-fouling species, a portion of the biofilm was scraped for morphological identification using a microscope (see section “Morpho-taxonomic analysis by microscopy”). Water samples (∼1 L) were collected from the surface water column (1.0 m depth) and placed into an autoclaved polycarbonate bottle on days 0, 15, and 30 of plate immersion, for each season. Scraped biofilm samples and sampled seawater were transported in ice coolers for subsequent DNA extraction. Microorganisms present in the water sample were concentrated using the PowerWater Sterivex DNA Isolation Kit (Qiagen, Hilden, Germany). Biofilms samples were preserved in LifeGuard Soil Preservation Solution (Qiagen, Hilden, Germany) and kept at −80°C until genomic DNA extraction was conducted. DNA from each biofilm sample was extracted and isolated using the Powersoil DNA Isolation Kit (Qiagen, Hilden, Germany) and stored at −20°C until sequencing. We used the PowerWater Sterivex DNA Isolation Kit for the recovery of DNA from microbial communities from marine water, and the PowerSoil Kit for the DNA extraction of biofilms since they have similar approaches for the amplification of DNA from environmental samples (Lear et al., 2018). Environmental parameters of the seawater were measured with a multiparameter sensor from HQ40D (Hach Lange, Berlin, Germany) – over the experimental period. Environmental parameters values were analyzed using the non-parametric Kruskal–Wallis test, followed by two-tailed Mann–Whitney–Wilcoxon test for pairwise comparisons also using R (version 3.6.3) (R Core Team, 2020). Statistical tests were performed for the environmental parameters after performing the Shapiro–Wilk normality test, as the data was shown not to be normally distributed.

### DNA Sequencing and Sequence Analysis

The 16S rRNA gene amplicon sequencing was performed by LGC Genomics (LGC Genomics GmbH, Berlin, Germany) using 300 bp paired-end reads, and sequencing the V3 region on an Illumina MiSeq sequencer (Illumina, Inc., San Diego, CA, United States) using the universal 16SrRNA bacterial primers 341F and 785R (Klindworth et al., 2013). All processing and data analyses were performed using the Quantitative Insights Into Microbial Ecology software (QIIME v.1.8.0) (Caporaso et al., 2010). In short, FASTQ files received were previously subjected to base calling and demultiplexing (Illumina bcl2fastq v.1.8.4). Pairs of forward and reverse primer/adapter-trimmed sequences were overlap-combined using BBMerge v.34.48. Reads with a final length of less than 100 bp were discarded, and the quality of paired-end Illumina reads was filtered based on quality (Phred) scores per-nucleotide using the quality parameters recommended by Bokulich et al. (2013) with a maximum unacceptable score of 20. The chimeric sequences were filtered out using UCHIME v.4.2.40. After quality filtering and chimera removal, a total of 2,961,008 sequences were obtained across all samples (average 44,863; minimum 495; maximum 269,867). Reads were assigned to OTUs using UCLUST (Edgar, 2010), clustering at 97% sequence identity. OTUs (Operational Taxonomic Units) were classified against the Greengenes (v.13.5) database (DeSantis et al., 2006), as implemented in the QIIME pipeline. Alpha-diversity indices (observed number of OTUs, Chao1, Shannon and Simpson) and beta-diversity (unweighted UniFrac) (Lozupone et al., 2007) were calculated using the phyloseq R package (v. 1.30.0) (McMurdie and Holmes, 2013), and the samples rarefaction curves were calculated with the vegan R package (v. 2.5.6) (Oksanen et al., 2016), both in RStudio (v. 1.1.453) (R Studio Team, 2015) and R statistical software (version 3.6.3) (R Core Team, 2020). For beta-diversity analyses the OTU table was transformed to even sampling depth by multiplying the OTU relative abundances by 10^6^.^1^ The variability in microbial composition was then examined using principal coordinate analysis (PCoA) conducted with the unweighted UniFrac distance matrix (Lozupone et al., 2007) with the phyloseq package (v. 1.30.0). An analysis of similarities (ANOSIM) between the microbial community composition, using the unweighted UniFrac dissimilarity matrix, with treatment, condition or season was tested with the *anosim()* function from the vegan package, using 999 permutations (Oksanen et al., 2019). The individual OTUs that contributed to the community differences observed with ANOSIM for treatment, condition or season were determined with a similarity percentage (SIMPER) analysis using the *simper()* function, with 100 permutations, from the vegan package. The data was analyzed in terms of OTUs, following a more traditional approach in order to compare more directly our results with previous marine biofilm studies based on OTUs. Although, adopting a strategy with ASVs (Amplicon Sequence Variants) (Callahan et al., 2017) could improve the comprehensiveness of the data, it was considered that microbial richness, diversity metrics, composition of dominant taxa as well overall ecological results would not vary among the different approaches, either OTU’s or ASVs (Glassman and Martiny, 2018). Statistical differences between the groups tested for alpha-diversity analysis, and the classification of chloroplast sequences were analyzed using the non-parametric Kruskal–Wallis test, followed by two-tailed Mann–Whitney–Wilcoxon test for pairwise comparisons also using R. These tests were applied after the Shapiro–Wilk normality test, as the data was shown not to be normally distributed. One-way ANOVA followed by Fisher’s unprotected LSD was used to determine significant differences for each taxon in each of the tested conditions (GraphPad Prism v.5, GraphPad Software Inc., La Jolla, CA, United States). Sequencing reads for this study were deposited in the European Nucleotide Archive (ENA) under the accession number PRJEB33600.

### Classification of Chloroplast Sequences

An additional OTU table was generated for OTUs classified as “chloroplast” against the Greengenes database (v.13.5) in the last day of sampling for both seasons (day 30) which correspond to phytoplankton. Since the taxonomic assignment of chloroplasts sequences against Greengenes was generally restricted to the Phylum or Class level, we used the BLASTn (Altschul et al., 1990) tool to classify chloroplast sequences using the NCBI reference sequence database (RefSeq version 89) in order to improve the taxonomic assignments following Needham and Fuhrman (2016). As chloroplasts are present over the phytoplankton life cycle (Decelle et al., 2015) and are highly variable among similarly sized organisms, we decided to perform a taxonomic assignment based on chloroplast 16S rRNA gene abundance, to provide a taxonomic assignment of the phytoplankton organisms present in our samples. Consequently, we classified the “chloroplast” sequences by performing a BLASTn search against the PhytoRef database (Decelle et al., 2015), a curated database of plastidial 16S rRNA gene sequences of photosynthetic eukaryotes (downloaded in July 2018). The most abundant taxon, characterized via sequence abundance, with classification via best match of the 16S rRNA genes of chloroplasts to NCBI and PhytoRef databases. Since the sequence identity of sequences alignments varied between the two databases, we established rules for determining which classification to use, following the protocol of Needham and Fuhrman (2016). Accordingly, if a chloroplast sequence had a NCBI match with <95% identity, and the PhytoRef match had >95% identity, we used the PhytoRef classification), otherwise the NCBI-derived assignment was used (see Supplementary Table S1).

### Morpho-Taxonomic Analysis by Microscopy

**FIGURE 1:**
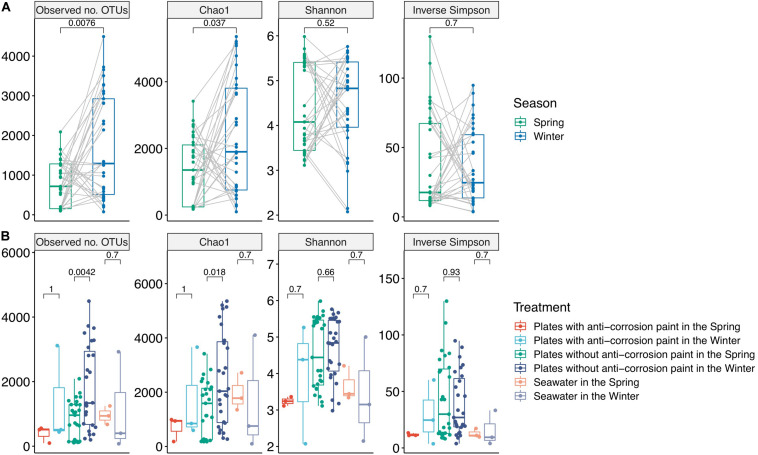
Alpha-diversity values for the total samples grouped by season: spring (*n* = 33) and winter (*n* = 33) **(A)**, and grouped by the presence or absence of anti-corrosion paint, and including the planktonic communities among the two different seasons **(B)**. Values represent differences among treatment groups using the two-tailed Mann–Whitney–Wilcoxon test.

To visually identify possible macrofouling species attached to the surfaces in the last of sampling of each season, a qualitative taxonomic analysis of the plates in the presence and absence of the paint was performed. Briefly, and independent 2 cm by 2 cm square of the colonizing community (see Figure 1) was scraped. The sample was kept in an icebox and transported to the lab where a morpho-taxonomic analysis was performed using a light microscope at 10×, 40×, and 100× magnifications. Photographs were obtained using a digital camera unit Olympus U-TV1X-2 DP72 attached to an Olympus SZX10 microscope or an Olympus BX41 phase-contrast microscope. Identification was performed to the lowest possible taxonomic level, by consulting reference taxonomic guides (Kraberg et al., 2010; Mees, 2015).

## Results

### Environmental Parameters

During the spring sampling (24th of May–23rd of June of 2016) water temperature values ranged between a minimum of 16.1°C and a maximum of 18.5°C, with an average temperature of 17.1°C ± 0.9°C (Table 1). Values of pH ranged between 7.09 and 7.67 with an average pH of 7.37 ± 0.20 (Table 1). For the winter sampling (7th February–8th March 2017) the water temperature values varied between a minimum of 13.3°C and a maximum of 13.9°C with an average temperature of 13.6°C ± 0.3°C (Table 1). The pH values during winter varied between 7.64 and 7.94 with an average of 7.88 ± 0.09 (Table 1). Regarding the evolution of biofilm biomass in the sampled days, during spring there was an average increase of 87.6 ± 3.1 grams per bare plate, and an increase of 80.2 ± 1.8 g per plate covered with corrosion paint. During the winter, there was an average increase of 83.3 ± 2.1 g per bare plate, and an average increase of 78.1 ± 1.1 g plate with anti-corrosion paint (see Supplementary Figure S2 for representative photographs of biofilm temporal succession for each day of season) Salinity values during our sampling varied between 28.9 PSU and 33.3 PSU with an average of 31.3 PSU, while the dissolved oxygen values were between 7.41 and 7.94 mg/L, with an average of 7.71 mg/L. The average daylength for spring was 14:58 (hours:minutes) while for winter it was 10:49 (hours:minutes). For the analyzed environmental parameters, only temperature and daylength values were significantly different between the seasons (Kruskal–Wallis test, *p* < 0.05).

**TABLE 1:** Environmental parameters at the Leixões Port for each of the sampled days.

Parameters spring	Day 1	Day 2	Day 4	Day 7	Day 10	Day 14	Day 21	Day 25	Day 30
pH	7.31	7.52	7.51	7.67	7.21	7.56	7.21	7.09	7.21
Temperature (°C)	16.1	16.5	16.6	17.5	17.5	18.2	18.6	16.3	16.5
PSU	31.1	31.2	29.6	29.8	32.5	30.9	31.5	29.7	30.2
Daylength (hours: minutes)	14:46	14:47	14:50	14:54	14:59	15:02	15:07	15:08	15:08

**Parameters Winter**	**Day 1**	**Day 2**	**Day 4**	**Day 7**	**Day 10**	**Day 14**	**Day 21**	**Day 25**	**Day 30**

pH	7.65	7.87	7.91	7.92	7.91	7.94	7.92	7.91	7.85
Temperature (°C)	13.3	13.6	13.6	13.3	14.0	13.5	13.9	13.9	13.5
PSU	30.7	31.3	33.3	32.5	33.0	32.9	29.3	31.7	32.1
Daylength (hours: minutes)	10:19	10:22	10:27	10:34	10:42	10:52	11:11	11:22	11:35

### Alpha- and Beta-Diversity

As part of the results of the first step of the bioinformatic workflow, the combined forward and reverse reads of the NGS data yielded a dataset comprised of 4,950,919 combined reads (154,784 reads or 3.03% of the total reads were unmerged read pairs). For the total of 66 samples an average 75,014 reads *per* sample was obtained (minimum 762; maximum 453,850 reads). All of the rarefaction curves for the winter samples and most of those for the spring samples reached a saturation level, suggesting that the sequencing depth *per* sample was generally adequate to cover the whole diversity of the biofilm and planktonic samples (Supplementary Figure S3). In our data, we did not observe a major difference in the alpha-diversity indexes among the entirety of biofilm and planktonic samples. The Chao1 metric varied between 193.8 ± 24.7 for the first day of spring and 4053.2 ± 1237.2 for the fourth day of winter, the lowest and highest values, respectively. We found an average Chao1 value for spring of 1387.5 ± 969.7 and for winter of 2332.4 ± 1729.3, with the average of plates with no paint of 1939.8 ± 1501.6, and for the plates with anti-corrosion paint of 1201.8 ± 1241.0. Regarding the alpha diversity and the unweighted differences among all sampling days, there was a significant lower diversity in the spring both in the observed OTUs (two-tailed Mann–Whitney–Wilcoxon test, *p*-value < 0.01) and for the Chao1 index (two-tailed Mann–Whitney–Wilcoxon test, *p*-value < 0.05) (Figure 1). Similarly, there was a lower diversity in the plates without anti-corrosion paint in the spring than in the winter both for the observed number of OTUs (two-tailed Mann–Whitney–Wilcoxon test, *p*-value < 0.01) and in the Chao1 index (two-tailed Mann–Whitney–Wilcoxon test, *p* < 0.05). This was observed for the spring sampling but not during winter (Figure 1).

**FIGURE 2:**
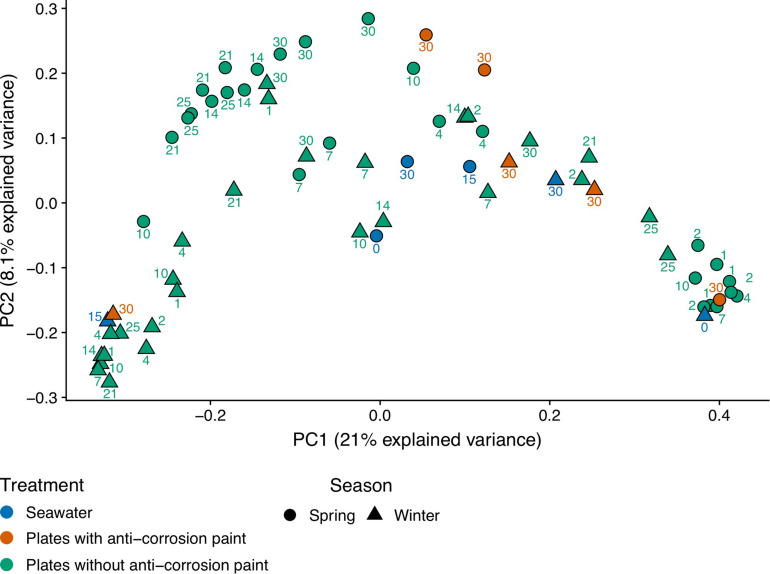
Principal coordinate analysis (PCoA) of unweighted UniFrac distances after transforming the bacterial 16S rRNA gene OTU table to even sampling depth. Circle and triangle shapes represent spring and winter seasons, respectively. The different colors highlight the treatments along each season: plates without anti-corrosion paint in spring and winter, plates with anti-corrosion paint in spring and winter, and planktonic communities in spring and winter.

Regarding the effect of the day of sampling, there was in average a lower value of diversity in the second day of sampling with an increase of diversity until day 25, followed by a decrease in the last day of sampling but without any statistically significant differences between those days. Nevertheless, there were major differences in the diversity values for the sampled days within seasons: during spring there was a progressive increase of diversity from the first day of sampling until the day 25 of sampling (observed number of OTUs, Chao1, Shannon and Inverse Simpson), followed by a decrease of diversity in the last day of sampling (day 30) (Kruskal–Wallis test, *p*-values < 0.01). During winter, we observed a different pattern, with an increase of diversity until the fourth day of growth, followed by a non-continuous and slight decrease, and another increase at the last days of sampling, indicating that there was still no stabilization of diversity. Despite these trends, there were no significant differences between the sampled days. The plates without corrosion paint had the highest total number of OTUs in both seasons, followed by seawater in the spring and by plates without corrosion paint in the winter (Supplementary Figure S4). For both seasons, the planktonic communities and the communities growing in the plates with anti-corrosion paint had the least OTUs in common (Supplementary Figure S4). Regarding beta-diversity, ANOSIM analysis demonstrated that the bacterial composition was affected more by the sampling type, plates with and without anti-corrosion paint or seawater (ANOSIM, *R* = 0.24 *p* < 0.01), followed by the interaction effect of sampling type and season (ANOSIM, *R* = 0.23; *p* < 0.01) and the season (ANOSIM, *R* = 0.12; *p* < 0.01), but less affected by the overall sampling day (ANOSIM, *R* = 0.07; *p* < 0.1). Furthermore, we found a distinct cluster composed of most of the early spring biofilm samples (Figure 2), suggesting that these spring pioneer communities are distinct both from later spring bacterial communities and also from almost the entirety of the winter samples.

### Taxonomic Temporal Succession

**FIGURE 3:**
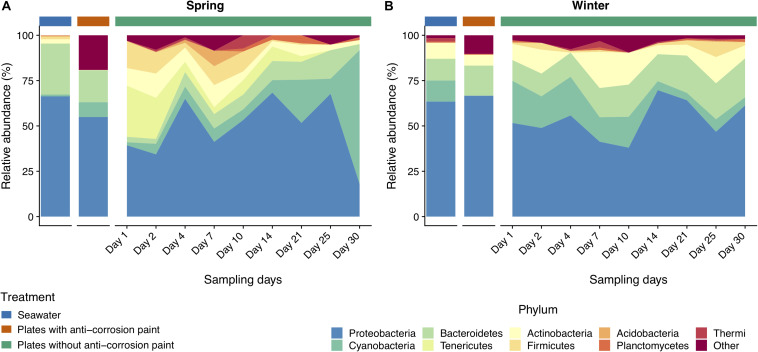
Taxonomic diversity and relative abundance at the phylum level of bacteria from the samples collected at spring **(A)** and at winter **(B)**. Sequences were taxonomically assigned to the Greengenes database (DeSantis et al., 2006) using QIIME (Caporaso et al., 2010). For clarity of presentation, unassigned sequences and phyla comprising < 1% of the total number of sequences within a sample were simply classified as “Other.”

Taking into consideration the totality of the samples, the bacterial communities were dominated by the phylum Proteobacteria (52.2 ± 21.3%), followed by Bacteroidetes (15.6 ± 5.1%), Cyanobacteria (14.9 ± 7.1%), and Actinobacteria (7.8 ± 2.3%) (Supplementary Table S2). Proteobacteria were strongly represented by Alphaproteobacteria (30.1 ± 0.7%), and Gammaproteobacteria (25.0 ± 5.6%) (Supplementary Table S3). Chloroplast (11.6 ± 3.7%), and Actinobacteria (7.2 ± 2.5%) were the subsequent most dominant groups classified at the class level in all samples (Supplementary Table S3). At the order level, Rhodobacterales were most abundant (15.7 ± 4.6%), followed by Stramenopiles (11.1 ± 4.3%), Flavobacteriales (8.7 ± 4.0%), Burkholderiales (7.8 ± 3.0%) and Actinomycetales (7.8 ± 2.6%) (Supplementary Table S4). Rhodobacteraceae (22.1 ± 2.3%) is by far the most abundant family in all the samples, followed by Flavobacteriaceae (10.9 ± 4.3%), Halomonodaceae (7.4 ± 2.7%), and Alteromonodaceae families (5.4 ± 3.9%) (Supplementary Table S5). Considering the genera, the most abundant were *Octadecabacter* (4.2 ± 1.1%); *Friedmanniella* (4.0 ± 2.2%); *Clostridium* (2.8 ± 2.4%); *Propionibacterium* (2.8 ± 0.9%*); Oleispira* (2.0 ± 2.4%), and *Pseudoalteromonas* (1.9 ± 1.3%) (Supplementary Table S6). Regarding taxonomic differences in samples grouped by the tested seasons, no major differences were found at the phylum level (Figure 3), except that Actinobacteria and Planctomycetes were more abundant in the entire winter sampling than during spring sampling (5.7 ± 2.2% compared to 1.9 ± 1.5%, and 1.1 ± 0.3% compared to 0.5 ± 0.2%, respectively (one-way ANOVA, LSD, *p*-values < 0.05) (Supplementary Table S2). On the other hand, the phylum Tenericutes was significantly more abundant in spring (0.5 ± 0.1%) than in winter (0.02 ± 0.01%) (one-way ANOVA, LSD, *p*-value < 0.001) (Supplementary Table S2).

**FIGURE 4:**
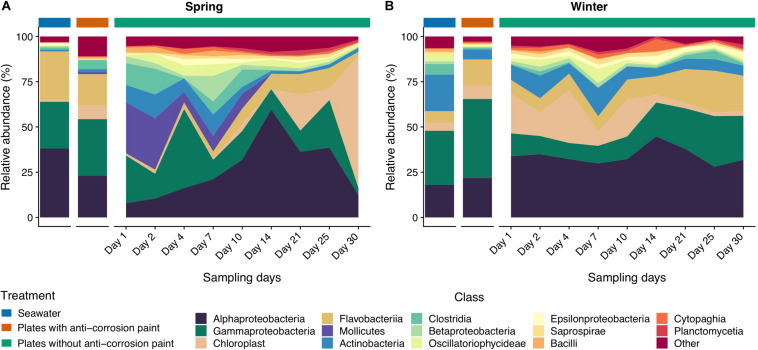
Taxonomic diversity and relative abundance at the class level of bacteria from the samples collected at spring **(A)** and at winter **(B)**. Sequences were taxonomically assigned to the Greengenes database (DeSantis et al., 2006) using QIIME (Caporaso et al., 2010). For clarity of presentation, unassigned sequences and classes comprising < 1% of the total number of sequences within a sample were simply classified as “Other.”

**FIGURE 5:**
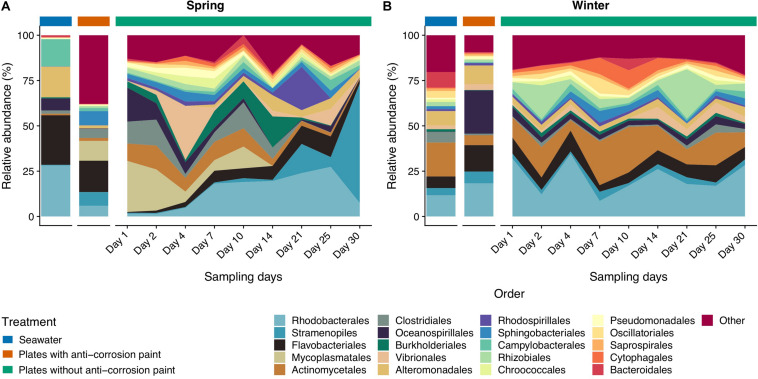
Taxonomic diversity and relative abundance at the order level of bacteria from the samples collected at spring **(A)** and at winter **(B)**. Sequences were taxonomically assigned to the Greengenes database (DeSantis et al., 2006) using QIIME (Caporaso et al., 2010). For clarity of presentation, unassigned sequences and orders comprising < 1% of the total number of sequences within a sample were simply classified as “Other.”

The classes Clostridia, Cytophagia and Mollicutes and the group comprising Chloroplast sequences were also significantly more abundant in all samples collected during spring than during the winter (Figure 4) (one-way ANOVA, LSD, *p*-values < 0.05) (Supplementary Table S3). The same was verified for the orders Burkholderiales, Clostridiales and Methylococcales (one-way ANOVA, LSD, *p*-values < 0.01), and for the families Alteromonodaceae and Clostridiaceae (one-way ANOVA, LSD, *p*-values < 0.01) (Figure 5). On the other hand, during winter there was a higher abundance of the orders Actinomycetales and Deinococcales (one-way ANOVA, LSD, *p*-values < 0.01), as well of the families Nocardioidaceae and Xenococcaceae (one-way ANOVA, LSD, *p*-values < 0.01 and <0.05, respectively) (Supplementary Tables S4, S5). The dominant genera *Friedmanniella*, *Hymenobacter*, *Sulfurimonas*, and *Proteus* were more abundant in spring than in winter, whereas in winter “*Candidatus* Portiera,” *Clostridium*, *Mycoplasma*, *Oleispira*, *Pseudoalteromonas* were more abundant (one-way ANOVA, LSD, all *p*-values < 0.05). The genera *Friedmanniella*, *Hymenobacter*, *Sulfurimonas Proteus* were more abundant in spring than in winter, while “*Candidatus* Portiera,” *Clostridium*, *Oleispira*, *Pseudoalteromonas*, *Proteus* were more abundant during winter than during spring (one-way ANOVA, LSD, all *p*-values < 0.05) (Supplementary Table S6).

As for the effect of the lifestyle (biofilm or planktonic) as well the effect of the presence or absence of anticorrosion paint on the taxa, at the phylum level, Acidobacteria and Actinobacteria were more abundant in seawater than in the sampled biofilms growing in surfaces with anti-corrosion paint. During spring, Tenericutes was much more abundant in steel surfaces with no anti-corrosion paint than in the collected seawater, or in steel plates covered with anti-corrosion paint (one-way ANOVA, LSD, *p*-values < 0.001) (Figure 3 and Supplementary Table S2). Regarding the taxonomic level class, Bacilli, Cytophagia and Epsilonproteobacteria were much more abundant in steel plates without paint (*p*-values < 0.01), where there was also a significantly higher abundance of the orders Cytophagales, Flavobacteriales, Oceanospirillales, Stramenopiles (Figure 4 and Supplementary Table S3). The orders Flavobacteriales, Methylococcales, and Stramenopiles, as well of the respective families Flavobacteriaceae and Vibrionaceae were more abundant on biofilms growing on the steel surfaces than in the planktonic water (Figure 5 and Supplementary Table S4), suggesting a tendency to be generally more abundant in the biofilm stage. Additionally, the families Clostridiaceae, Flavobacteriaceae, Phormidiaceae, Vibrionaceae were more abundant on steel surfaces without paint (Supplementary Table S5). This is particularly true for the genus *Mycoplasma* (one-way ANOVA, LSD, *p*-value < 0.001), as well of the genera *Loktanella* and *Sulfurimonas* (one-way ANOVA, LSD, *p*-values < 0.01) (Supplementary Table S6). On the other hand, Alteromonadales (as well the Alteromonodaceae family), Deinoccocales (and the Deinococcaceae family*)* and the family Xenococcaceae were much more abundant in the sampled planktonic communities than in the sampled biofilms either with or without paint (one-way ANOVA, LSD, *p*-values < 0.01, except for Alteromonadales, *p*-value < 0.001) (Supplementary Tables S4, S5). The same was observed for the genera “*Candidatus* Portiera,*” Chroococcidiopsis, Friedmanniella, Phormidium*, and *Sphingomonas* which were significantly more abundant in the planktonic communities than in the sampled biofilms (Supplementary Table S6).

Regarding taxonomic differences *per* day of the combined seasons, no major differences were found for most taxa, suggesting that the biofilm day of growth is generally not a major factor influencing taxonomic differences. At the phylum level the only exceptions were Acidobacteria which seems to be more abundant in early biofilm days (between days 2 and 14) (Figure 3 and Supplementary Table S2). Firmicutes were also more abundant in early days in spring than during winter, as well in the last days. For the other taxa there was also an increase of relative abundance along the different days of biofilm growth, such as the classes Sphingomonodales, Rhodobacterales (and the Rhodobacteraceae family), Vibrionales (and the Vibrionaceae family), as well the families Alteromonodaceae, Flavobacteriaceae, Halomonadaceae and Clostridiaceae, however this increase was not always progressive (Supplementary Tables S3, S4). The dominant Rhodobacteraceae family had a significant lower abundance in the first day of biofilm (0.7 ± 0.6%) (one-way ANOVA, LSD, *p*-value < 0.001), starting to dominate in the second of sampling until reaching a maximum in the last day (15.3 ± 7.9%) (Supplementary Table S5). Proteobacteria and Cyanobacteria were more abundant since day 14 until the last sampling day during spring, being much more abundant in spring than during winter, particularly in the last day of sampling (one-way ANOVA, LSD, *p*-values < 0.001) (Figure 3 and Supplementary Table S2). The dominant classes Alphaproteobacteria and Flavobacteria were more abundant in the intermediate days of the spring biofilm, while other classes that dominated spring biofilms in the later days include Chloroplast and Planctomycetia (one-way ANOVA, LSD, *p*-values < 0.01) (Figure 4 and Supplementary Table S3).

**FIGURE 6:**
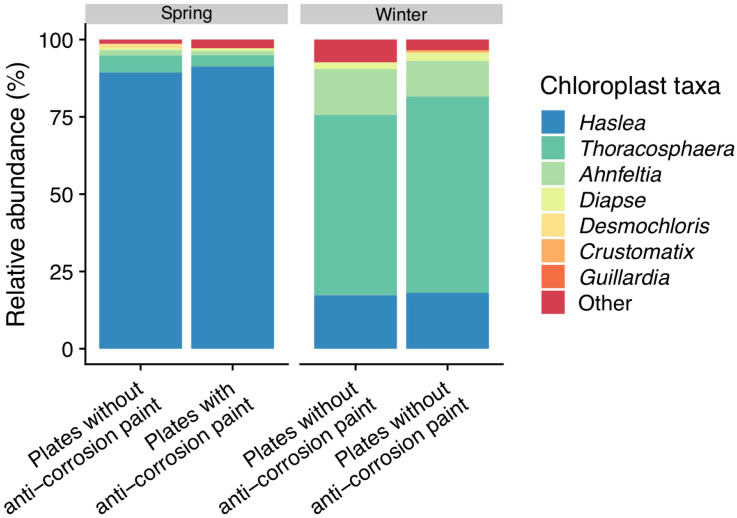
Taxonomic diversity and relative abundance of sequences classified by Greengenes as “chloroplast” at the last day of sampling (day 30). Sequences were classified by the NCBI and PhytoREF databases at the lowest possible taxonomic ranks and chosen based on the best match. For clarity of presentation, unassigned sequences comprising < 1% of the total number of sequences within a sample were simply classified as “Other.”

The blast analysis of “Chloroplast” sequences against NCBI and phytoREF databases (see Supplementary Table S1) determined that on the last day of spring sampling the phototrophic communities were dominated by the diatom *Haslea* sp. with no significant difference found between the surfaces with and without anti-corrosion paint. Furthermore, there was also a low abundance of the red alga *Ahnfeltia* (without significant differences between with or without anti-corrosion paint) and the green algae *Desmochlorosis* which was less abundant in the presence of the anti-corrosion paint (one-way ANOVA, LSD, *p*-value < 0.05). On the winter sampling, a dominance of the dinoflagellate *Thoracosphaera* was observed, followed by the low abundance of the diatom *Haslea* sp., without any significant differences for the biofilms growing with and without anti-corrosion paint, in terms of the most abundant taxa (Figure 6). The morphological identification by microscopy, also revealed the colonization of microeukaryotic organisms, particularly of diatoms and microalgae in the last day of sampling for both seasons (see Supplementary Figure S5). In the last day of spring sampling (day 30) macrofouling algae were also observed by direct observation of the steel plates (see Supplementary Figure S2), and subsequently through microscopic taxonomic identification. On the other hand, macrofouling colonizers were not observed colonizing the plates in last days of winter (see Supplementary Figures S2, S5).

## Discussion

### Temporal Alpha- and Beta-Diversity Patterns

Biofilms and planktonic communities are known to present dissimilar communities, with the latter often being associated with higher diversity values than the former (Jones et al., 2007; Lee et al., 2008; Shikuma and Hadfield, 2010). When characterizing the dynamics of marine microbial communities, some authors (Gilbert et al., 2012; Cram et al., 2015) found distinct cyclic alpha-diversity patterns with a maximum in winter and a minimum in summer with seasonal differences tending to be greater than inter-annual changes (Fuhrman et al., 2015). Compared to planktonic communities, surface-associated microorganisms normally possess compositional variations and dynamics responding to seasonal, daily or even hourly variations (Ghiglione et al., 2007; Grossart, 2010). We also found an average maximum of diversity in the winter samples which is contradictory with some previous biofilm studies which did not show clear seasonal diversity patterns (Patil and Anil, 2015), but similar to a study which also presented highest diversity during winter (Elifantz et al., 2013). The average increase of the alpha diversity of all observed indexes during spring may suggest that during spring there was a transition to mid-stage biofilms, which have moderate to low diversity when compared to early biofilms, and this relatively lower diversity may be explained by competitive species dominating the biofilms or by variations of temperature found during the sampling period (Jackson et al., 2001). This confirms the notion that biofilm development and diversity are interconnected, and diversity is dependent on the testing time (Jackson et al., 2001), and our results seem to suggest that they are also dependent on the season. Regarding the beta-diversity analysis we observed that the bacterial diversity (bacterial community composition) was more influenced by the seasonal day of biofilm growth, with a PCoA analysis clearly separating most early spring biofilm samples from all other samples (Figure 2). Regarding the application of the anti-corrosion paint, during the spring sampling, it resulted in a lower diversity compared with biofilms growing without paint (one-way ANOVA, LSD; *p*-value < 0.05). This is in accordance with previous studies demonstrating that antifouling paints significantly influence the bacterial diversity of biofilms grown on them (Muthukrishnan et al., 2014; Briand et al., 2017). However, this effect was not observed during winter, which suggests that the effect of this paint on the bacterial diversity may be also dependent of seasonal variations, such as temperature or daylength.

### Temporal Succession of Marine Biofilms in Steel Plates

In this study, the most abundant phylum was Proteobacteria (Figure 3), in line with the existing body of work that points out that Proteobacteria, particularly the class Alphaproteobacteria dominate marine biofilms in different settings (Dang and Lovell, 2000; Huggett et al., 2006; Jones et al., 2007; Chung et al., 2010). Proteobacteria are known to be pioneer surface colonizers (Dang et al., 2008, 2011; Slightom and Buchan, 2009) and their abundance was reported across a range of different environmental conditions (Liu et al., 2015; Qi et al., 2016) and in seawater (Suh et al., 2015; Mancuso et al., 2016). Among the Alphaproteobacteria class, there was the dominance of the order Rhodobacterales and of the Rhodobacteraceae family, similar to previous studies involving marine biofilms growing on steel surfaces (Emerson et al., 2010). The Alphaproteobacteria class is known to survive well in oligotrophic environments (Alonso and Pernthaler, 2006), as is the case of initial phase of biofilm formation, when the amount of nutrients and organic carbon close to the surface is limiting (Flemming, 2002). Bacteroidetes were the second most abundant phylum in our samples, followed by Cyanobacteria. Within the phylum Bacteroidetes, the dominance of the order Flavobacteriales in the biofilm samples was observed, which is in line with past studies sampling marine biofilm in temperate waters (Dang et al., 2011; Pollet et al., 2018). In contrast to Alphaproteobacteria, Bacteroidetes require higher levels of nutrients and organic matter (Kirchman, 2002; Elifantz et al., 2013). Bacteroidetes are well-known for colonizing steel surfaces in the marine environment and to be associated with biological corrosion (Kirchman, 2002). Members of this group are efficient surface colonizers and can take some advantage of primary production by chemolithotrophic Fe- and S-oxidizing bacteria during biofilm formation (Doghri et al., 2015).

Regarding temporal variations, we observed important major seasonal changes of the early surface colonizers. In the first 4 days of spring, we observed a strong presence of Gammaproteobacteria and also of Mollicutes. In contrast, during the winter sampling period, Alphaproteobacteria were much more abundant in the first days of sampling, with a lower abundance of Gammaproteobacteria and a complete absence of the Mollicutes class (Figure 4). The overall dominance of the orders Rhodobacterales (Alphaproteobacteria) and Flavobacteriales (Bacteroidetes), however, did not seem to be affected by temporal variations (Figure 5), which corroborates the dominance of those taxa among marine biofilms sampled in different locations and sampling periods (Salta et al., 2013; Antunes et al., 2018). Overall, among Gammaproteobacteria, the genus *Oleibacter* strongly dominated microbial communities, something which had been previously been reported (Pollet et al., 2018). We also observed an early strong presence of *Clostridium* (Firmicutes), *Friedmanniella* (Actinobacteria), *Sphingomonas* (Alphaproteobacteria) and *Sulfurimonas* (Epsilonbacteria) (see Supplementary Table S6), which were the other main pioneer bacteria genera in our samples (particularly until day 4), and have not been commonly described in previous marine biofilm studies. On the other hand, later biofilms had a higher presence of *Propionibacterium* (Actinobacteria), while *Loktanella* (Alphaproteobacteria), *Octadecabacter* (Alphaproteobacteria) and *Pseudoalteromonas* (Gammaproteobacteria) were more abundant in the later days of biofilm growth. All these last taxa seem to be the dominant primary surface-colonizing bacterial groups in different locations at relatively later biofilm sampling days (Dang et al., 2008; Dang and Lovell, 2016). Similar to previous studies characterizing marine biofilms (Li et al., 2017; Pollet et al., 2018) there seems to be significant differences at the genus level as a response to the effect of seasonal variations, with more significant differences at the genus level than at higher taxonomic ranks. Similarly, the SIMPER analysis (Supplementary Table S7) also demonstrated that the taxa most contributing to the dissimilarity between different seasons were genera such as *Mycoplasma*, *Friedmanniella*, *Clostridium*, and *Propionibacterium*.

In general, the observed temporal variations suggest that marine biofilms sampled in the first days of colonization may be strongly dependent on seasonality. These were conspicuously distinct from all other biofilm samples (see Figure 2, and the ANOSIM analysis results). However, after day 7 of sampling, biofilm communities in both seasons appear to converge toward a similar composition (Figures 4, 5). Cyanobacteria are known to be dominant in biofilms reported from eutrophic areas (Muthukrishnan et al., 2014; Sanli et al., 2015). Despite the presence of these taxa being on average higher in spring than in winter, probably as a consequence of the significant higher temperature or daylength (see Table 1), the differences in our dataset were not significant. Gram-positive bacteria in our samples were mainly represented by two phyla, Actinobacteria and Firmicutes, the former was significantly more abundant in winter (Figure 3). In our results, there was a relatively lower abundance of Firmicutes compared to previous reports (Dang and Lovell, 2002), however, Firmicutes abundance was high on the first 10 days of winter biofilms (above 10%), while its relative abundance remained under 1% in the same period in the spring sampling. Therefore, the abundance of this taxon seems to experience a strong effect of seasonality. Another important taxonomic shift was observed on the last day of spring sampling (day 30), which refers to the group of Chloroplast sequences (eukaryotic phytoplankton) (average of 70.2%), a shift which was not observed during winter for the same period (average abundance was lower than 2%) (Figure 4). This shift toward phytoplankton communities could be confirmed by direct observation of the plates at the last day of spring sampling and by microscopic observation (Supplementary Figures S2, S5). These results suggest that seasonal changes in environmental variables may be more important than other interactions like competition or trophic interactions among biofilms (Pollet et al., 2018). When mentioning “seasonal” patterns however, it should be considered that there is an annual repeating pattern and not changes over a period of a few months (Fuhrman et al., 2015). Therefore, future studies should sample biofilms over different seasons for several years to confirm if there is a real seasonal pattern as at least partially suggested by our results, and not a simple monthly or occasional taxonomic change. Additionally, in our study, we detected significant differences between the average temperature and daylength of the two sampled seasons (see Table 1) which could have influenced the observed taxonomic shifts. In the generality of aquatic systems, temperature plays an important role in the changes of most biofilm propagation and metabolism (Bott, 1996; Rao, 2010).

### Taxonomy of Biofilms on Plates With Anti-corrosion Paint and of Planktonic Communities

In this study we employed a non-fouling release paint, and the dominant taxa were Alphaproteobacteria, Gammaproteobacteria, Firmicutes, and Bacteroidetes, similar to previous studies describing marine biofilm communities in fouling-release biocide-based coatings (McNamara et al., 2009; Chen et al., 2013). Fouling- release coatings revealed a significant effect on the phylum Proteobacteria and Bacteroidetes growth (Winfield et al., 2018), and another study demonstrated a sensitivity of Bacteroidetes and Actinobacteria to coatings based on pyrithione (Briand et al., 2017). In our study, however these most abundant phyla were not influenced by the applied anti-corrosion Intersheen 579 paint. Furthermore, in a recent study on microbial community succession over mild steel, the dominance of Proteobacteria and Bacteroidetes were described in different conditions, with the oxidizing iron group, the class Zetaproteobacteria within the Proteobacteria being the most abundant (Rajala et al., 2015; McBeth and Emerson, 2016). We also observed similar results, except that, in our study, the Zetaproteobacteria class had a low abundance (always lower than 0.1%). Among the Deltaproteobacteria class, the order Desulfobacterales, an order particularly associated to corrosion of artificial surfaces (Korenblum et al., 2010; Li et al., 2016; Tian et al., 2017) was also more abundant on the biofilm growing in steel surfaces (with paint and without paint) but it was not detected in the seawater, suggesting no significant effect of the anti-corrosion paint. This in contrast with previous studies in which copper-based biocidal coatings significantly inhibited the abundance of Deltaproteobacteria (Chen et al., 2013). These taxa, like the Clostridia class, produce acetic, butyric, or formic acids and their presence may also lead to corrosion of steel surfaces (Broda et al., 2000). Our results seem to suggest that these corrosion-associated taxa are particularly dependent on biofilm lifestyle. The particular importance of phylum Firmicutes growth on steel surfaces is due to the fact that some members of this phylum generate H_2_S and organic acids which can result in corrosion of surfaces (Paarup et al., 2006). In our study, comparing the plates with and without anti-corrosion paint, the class Clostridia was the second most abundant taxa growing in our steel surfaces, a class which is known to facilitate corrosion development in steel plates (Muthukrishnan et al., 2014; Tian et al., 2017). In the plates with paint, its barrier protection may explain the significant lower abundance observed for this taxon. In our study, the genus *Clostridium* was also more abundant in plates with no anti-corrosion covering and was particularly abundant in the early spring days (10.4% ± 4.3% in the first day and 13.5% ± 2.3% in the second day). The SIMPER analysis (Supplementary Table S7) also demonstrated that *Clostridium*, as well the genera *Halomonas, Mycoplasma*, and *Polaribacter* were some of the taxa most contributing to the dissimilarity between plates with and without paint. The Acetobacteraceae family (Alphaproteobaceria class, Rhodospirillales order), are known to produce acetic acid which can, in turn, corrode steel (Paarup et al., 2006). This family was less abundant on plates with anti-corrosion paint Intersheen 579, suggesting a possible role of this paint in controlling the growth of some steel corrosion associated taxa. The orders Alteromonadales, Oceanospirillales, Pseudomonadales, and Vibrionales were previously described as the main taxa responsible for biocorrosion on carbon steel (Xu et al., 2017), and in this work we observed a high abundance of these taxa since the very early days of surface colonization and with no major effect of the anti-corrosion paint. Similar results were observed in a previous study in which surfaces exposed to triclosan had no significant effect on the growth of Alteromonadales, Oceanospirillales (Eriksson et al., 2018). On the other hand, the use of surfaces containing copper influenced the growth of Alteromonadaceae (Briand et al., 2017) and of Flavobacteria (Muthukrishnan et al., 2014) which were not observed in our results, as the abundance of these taxa was not affected by the application of the paint. The observed results could also be influenced by color differences between the plates with and without anti-corrosion paint (see Supplementary Figure S1), as it is known that the color of the substrate may influence the formation of micro and macrofouling communities (Dobretsov et al., 2013), however we did not directly test this effect in our study.

Furthermore, we observed different patterns between biofilms and planktonic communities in both seasons. Specifically, in our results, the majority of the planktonic bacteria were affiliated with Alphaproteobacteria, Bacteroidetes, Cyanobacteria, and Actinobacteria taxa, similar to previous results describing planktonic communities on temperate waters (Giovannoni and Stingl, 2005) (Figures 4, 5). It has been proposed that biofilm and planktonic communities remain distinct even from early stages of biofilm formation but as the biofilm matures, its composition tends to become progressively more similar to the local microbial community (Ward et al., 2003; Pepe-Ranney and Hall, 2015). We observed a similar taxonomic shift with a progressive increase of Proteobacteria abundance (particularly Gammaproteobacteria). Marine biofilm communities are selected from planktonic communities but appear to be dissimilar from them (Lee et al., 2008; Briand et al., 2012; Dobretsov et al., 2018). These differences could also be due to the distinct structures between the complex and organized cellular of biofilms and of individual, planktonic cells which are present in the water column (Doghri et al., 2015). However, it is unclear if the community structure of biofilms on artificial surfaces differs from free-living bacteria because artificial surfaces and free-living bacteria have not often been examined together. Furthermore, we used different DNA extraction kits for the biofilm and planktonic communities which could result in the overestimation or underestimation of some taxa (Walden et al., 2017), however, these kits seem to have similar isolation efficiency in some previous studies (Tsuji et al., 2019).

### Detection of Possible Pathogenic Species and Identification of Macrofouling and Microeukaryotic Colonizers

In our sampling, the abundance of the genus *Vibrio* was higher in the winter sampling than during the spring sampling (one-way ANOVA, LSD, *p*-value < 0.05) which is surprising considering the optimum growth of this taxa is at higher temperatures (Eiler et al., 2006). The genus *Mycoplasma* (phylum Tenericutes, class Mollicutes) was particularly abundant in early spring biofilms (28.1% in the first day and 22.7% in the second day) being almost entirely absent in any of the winter sampling days, as well in spring sampling after day 4 (always lower than 0.1%) (see Supplementary Table S6). *Mycoplasma* are facultative anaerobes which are known to be associated with homeothermic animals and can also be human respiratory pathogens (Jacques et al., 2010). However, *Mycoplasma* were not described in previous *in situ* marine biofilm studies, and their relative high presence in our samples further demonstrates the role of marine biofilm communities as vectors of potential health hazardous taxa. The presence of these taxa maybe explained by the proximity of our sampling location to the River Leça (see Supplementary Figure S5), which can be a source of water contamination. A growing number of human pathogens have been reported to be present in marine biofilm communities (Ortega-Morales et al., 2007) and these microorganisms have been described as competitive, opportunistic and rapid colonizers, which can explain their recurring presence in young biofilms (Brinkmeyer, 2016). It should be noted that taxonomic assignment was only possible to conduct to the genus level, so it was not possible to determine the presence of pathogenic species in our samples such as the ones corresponding to the sequences classified as belonging to the *Vibrio* genus. Nevertheless, our results suggest that is important to identify possible pathogenic species in future biofilm studies in artificial substrates, particularly in port environments or marine environment with possible access to different sources of contaminations.

Regarding the microeukaryotes present in biofilms identified by microscopic observation and BLASTn analysis of the “Chloroplast” sequences, we found a high abundance of diatoms in our samples (see Figure 6 and Supplementary Figure S5). Diatoms are key members of biofilm communities as they are the main microphytobenthic group in marine epipelagic zones, especially in temperate areas (Cassé and Swain, 2006; Hunsucker et al., 2014). Diatoms colonizing surfaces are believed to have relative low diversity and only species from about 8–10 genera have commonly been documented to be problematic biofoulers of the ‘niche’ habitats provided by modern antifouling and fouling-release coatings (Molino and Wetherbee, 2008), most of them being pennate diatoms (Mitbavkar and Anil, 2008; Zargiel et al., 2011; Muthukrishnan et al., 2017). Some past studies did not show any major differences on the colonization rates on the tested artificial surfaces both on temperate and tropical locations after 2 weeks of submersion (Molino et al., 2009) and diatom colonization of tested surfaces did not present any clear temporal patterns (Patil and Anil, 2005; Elifantz et al., 2013). In our results however, we found a strong dominance of the motile pennate diatom *Haslea* sp. (over 80%) in the last day spring sampling (Figure 6), a genus which is known to colonize artificial surfaces (Winfield et al., 2018), while it had a much lower presence during winter (lower than 5%). The higher abundance of these phototrophic microorganisms could be explained by the significantly longer daylength during spring (see Table 1). The presence of *Haslea* sp. did not seem to be modulated by the application of the anti-corrosion paint (which was not reported as a biocide), differently from previous studies in which biocidal anti-fouling coatings significantly inhibited the settlement of certain genera of diatoms (Camps et al., 2014; Muthukrishnan et al., 2017). During winter, there was a high abundance of the phototrophic, calcareous dinoflagellate *Thoracosphaera* among all the chloroplast sequences, and a much lower abundance of diatom colonization when compared to spring (Figure 6). Low water temperatures are known to influence negatively the phototrophic community abundance, and diatom abundance is higher in warmer seasons, particularly as a part of spring or summer blooms (Molino et al., 2009), while *Thoracosphaera* is known to thrive at relatively lower water temperatures (Vink et al., 2003). Furthermore, the appearance of the green algae *Desmochloris*, and of the red macroalgae *Ahnfeltia* during the last day of spring sampling (see Supplementary Figures S2, S5), also suggests these fouling communities growing on the panels are moving beyond the initial stages of microfouling and are entering the first stages of macrofouling, while the winter communities remained dominated by heterotrophic prokaryotes.

## Conclusion

In conclusion, our study revealed significant distinct taxonomic profiles and diversity patterns among the bacterial communities of biofilms sampled in the two different seasons, suggesting that pioneer bacterial biofilm communities may face significant seasonal variations in the same location. These differences were more evident during the first 4 days of biofilm growth. Notable differences in microbial compositions were also observed in biofilms developed at different seasons compared to the planktonic communities which support the dynamic nature of these biofilms. Additionally, our results suggest that taxa associated to the corrosion of the steel surfaces were also detected among the colonizing bacteria and their abundance may also be dependent on the sampling day and season. Therefore, longer-term with different anti-biofilm/anti-fouling products with different mechanisms of action should be conducted to more adequately test their inhibitory effect in controlling bacterial biofilm communities in different temporal settings. It also should be also analyzed if this occurs with other artificial substrates immersed in marine environments. Besides employing NGS data, other techniques such as three-dimensional confocal fluorescence or *in situ* hybridization microscopy of labeled community members could help discern the temporal evolution and maturation of the attached biofilm communities and their abiotic components (e.g., exopolysaccharides) in the different seasons in future studies. Overall, our results should be taken in consideration for the management of biofilm growth on submerged structures in seaports, particularly of corrosion associated and possible pathogenic bacteria. Additionally, field studies should be conducted in several and consecutive seasons to fully infer if bacterial communities in marine biofilms respond mostly to daily/seasonal variations, or to more specific/broader temporal patterns.

## Data Availability Statement

The datasets generated for this study can be found in the European Nucleotide Archive (ENA) under the accession number PRJEB33600.

## Author Contributions

JTA, PL, and VV conceptualized and designed the work. JTA, AS, JA, AR, and PL worked on the acquisition, analysis or interpretation of data, and provided the approval for publication of the content. JTA, PL, and VV drafted the work and revising it critically.

## Conflict of Interest

The authors declare that the research was conducted in the absence of any commercial or financial relationships that could be construed as a potential conflict of interest.

## Footnotes

1 https://joey711.github.io/phyloseq/

## Supplementary Material

FIGURE S1 Sampling site: **(a)** Location of the Cruise Terminal of the Port of Leixões in Northern Portugal, Europe; **(b)** Marina floating platform; **(c)** Biofilm sampling structure.

FIGURE S2 Steel plates without and with anticorrosion paint before (day 0) and after the different days of biofilm growth (day 1 to day 30 for plates without paint and day 30 for plates with paint) and before scraping of biofilms corresponding both seasons (Spring and Winter).

FIGURE S3 Rarefaction plot based on number of OTUs for all samples.

FIGURE S4 Venn diagram for the total OTUs for the different treatments for Spring **(A)** and for Winter **(B)**.

FIGURE S5 Macrofouling and phytoplankton species identified through microscopic observation in the last day of sampling of both seasons.

TABLE S1 Blast-hit results used for the taxonomic assignment of “Chloroplast” sequences. All blast hit results comes from the search of Greengenes Chloroplast sequences in NT-NCBI and PhytoRef databases.

TABLE S2 Taxonomic abundance at the phylum level for the different treatments.

TABLE S3 Taxonomic abundance at the class level for the different treatments.

TABLE S4 Taxonomic abundance at the order level for the different treatments.

TABLE S5 Taxonomic abundance at the family level for the different treatments.

TABLE S6 Taxonomic abundance at the genus level for the different treatments.

TABLE S7 SIMPER analysis showing the taxa most contributing to total dissimilarity of bacterial communities colonizing surfaces and the respective contribution to the dissimilarity in the different treatments.
