# Supplementary figures and images for: Distinct Temporal Succession of Bacterial Communities in Early Marine Biofilms in a Portuguese Atlantic Port
Source: Front Microbiol. 2020 Aug 11;11:1938. doi: 10.3389/fmicb.2020.01938 (PMC7432428; doi:10.3389/fmicb.2020.01938)

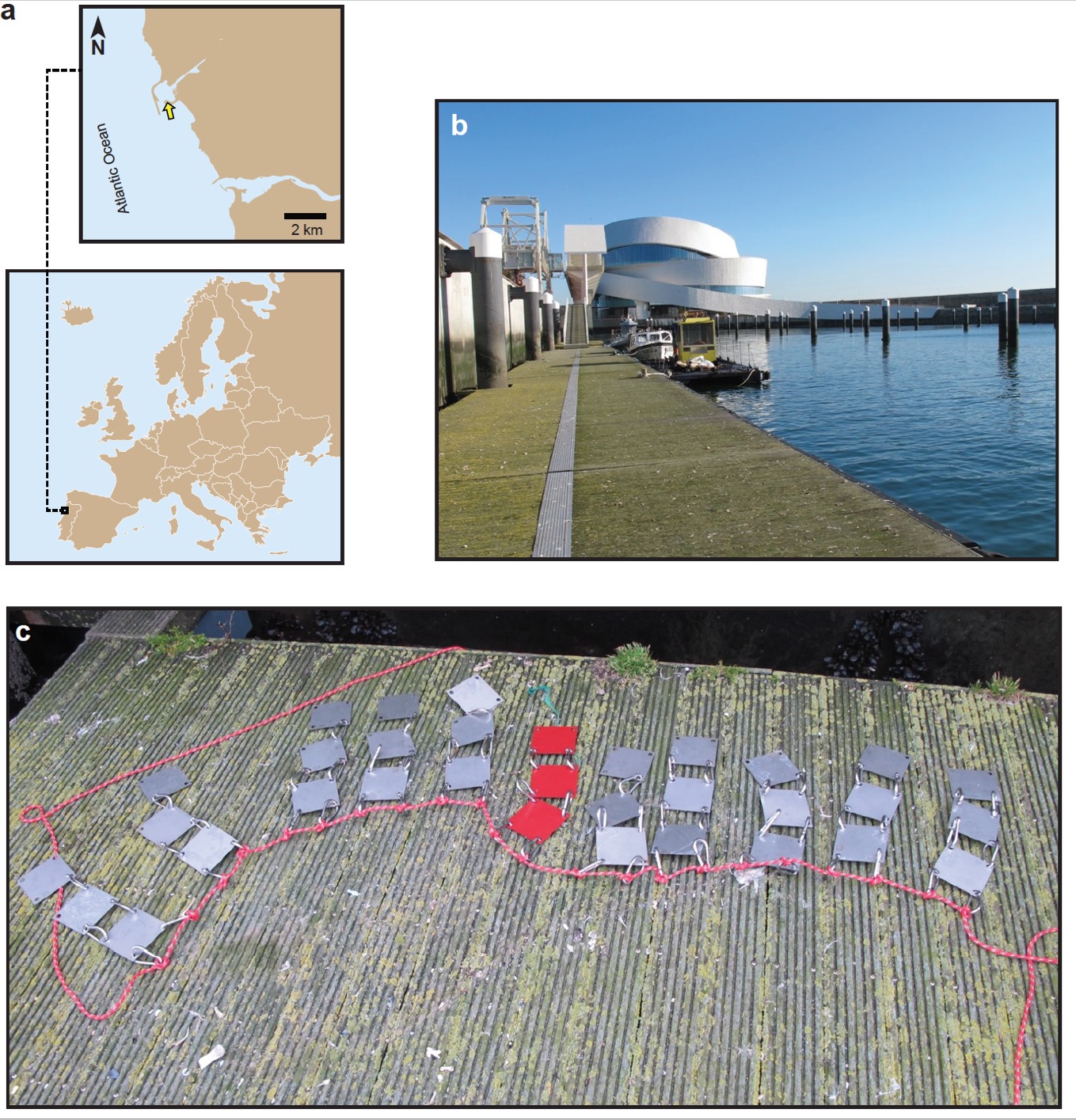

Supplement: FIGURE S1 — Sampling site: (a) Location of the Cruise Terminal of the Port of Leixões in Northern Portugal, Europe; (b) Marina floating platform; (c) Biofilm sampling structure. [file Image_1.jpeg]

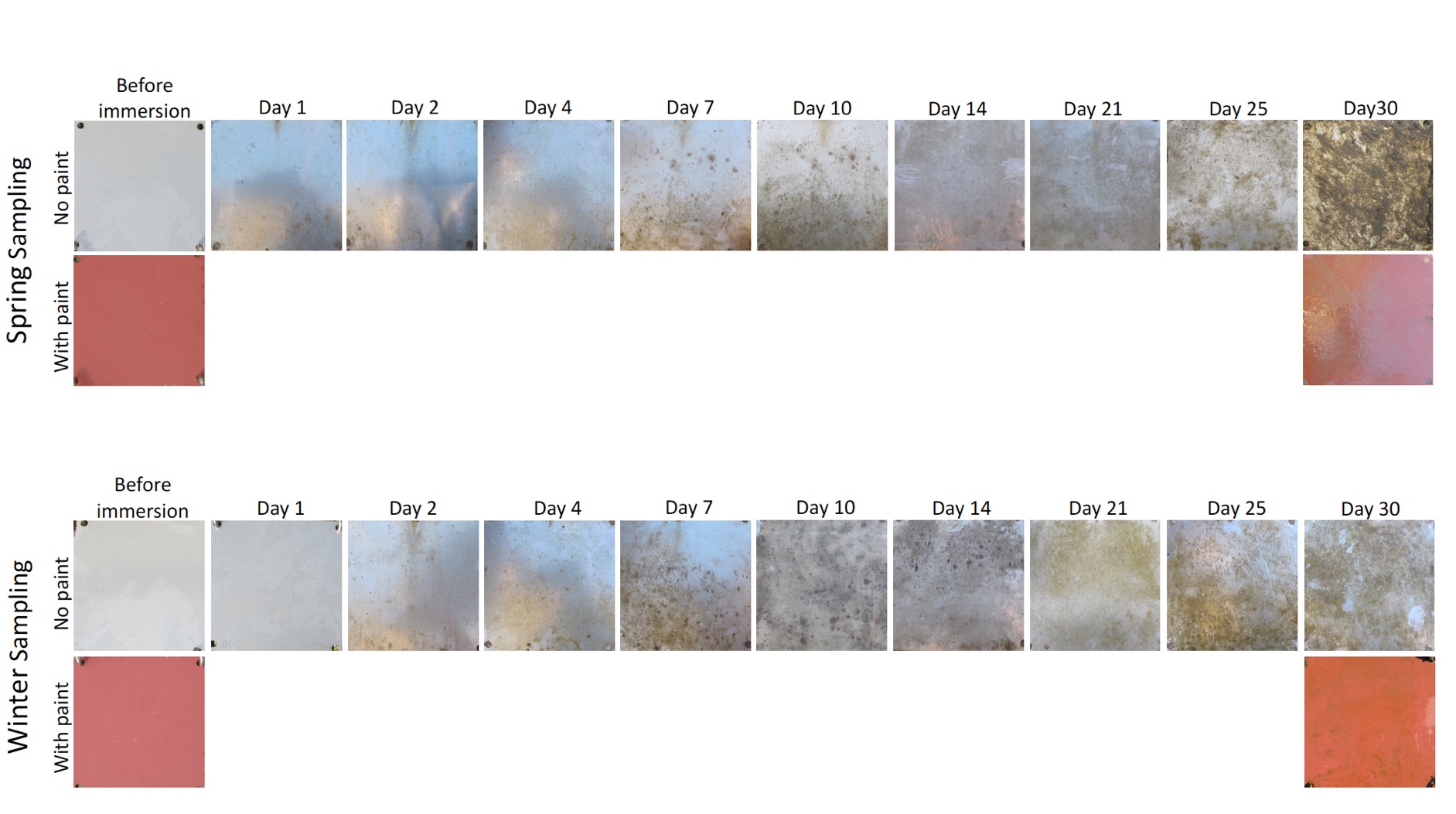

Supplement: FIGURE S2 — Steel plates without and with anticorrosion paint before (day 0) and after the different days of biofilm growth (day 1 to day 30 for plates without paint and day 30 for plates with paint) and before scraping of biofilms corresponding both seasons (Spring and Winter). [file Image_2.jpeg]

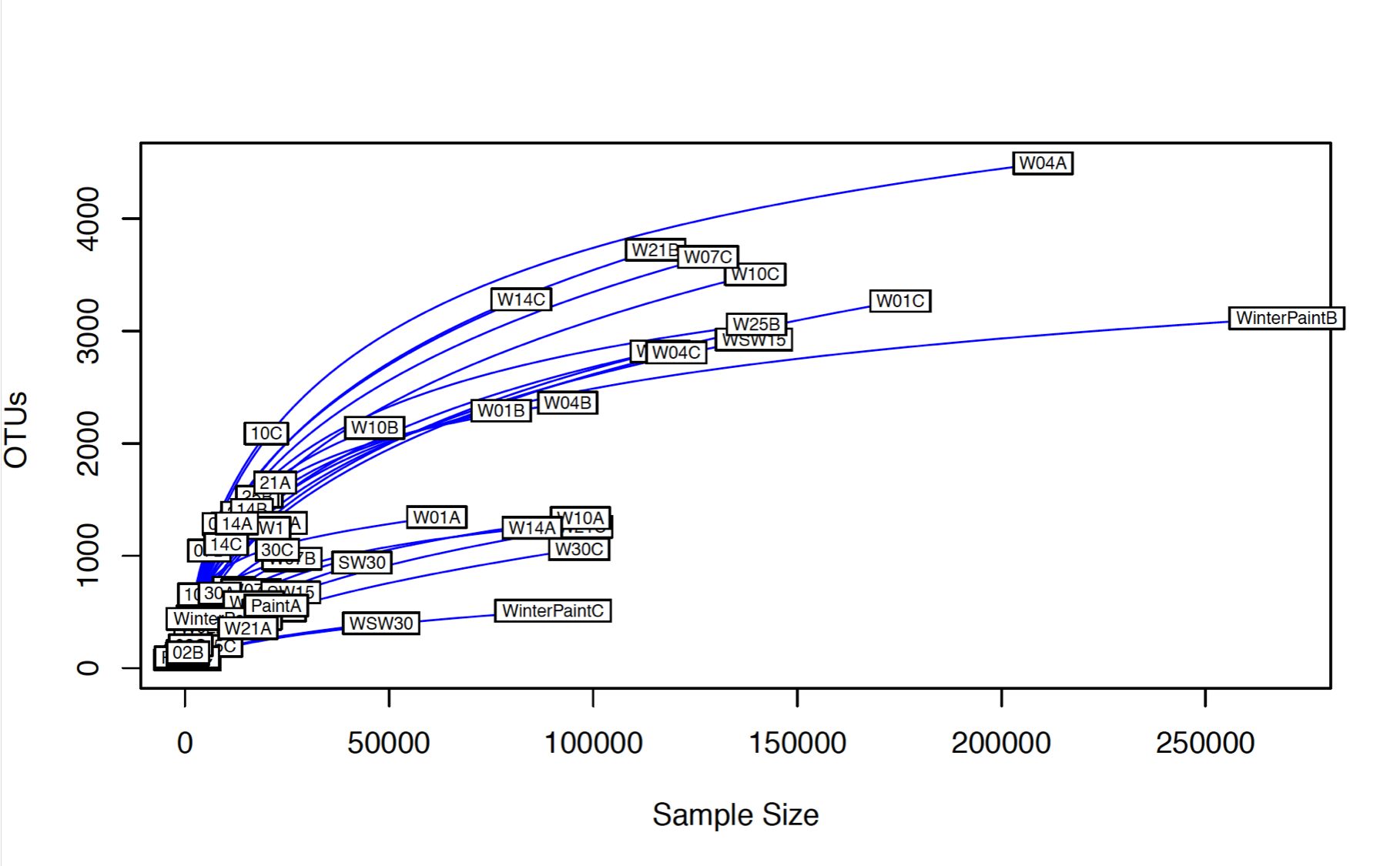

Supplement: FIGURE S3 — Rarefaction plot based on number of OTUs for all samples. [file Image_3.tiff]

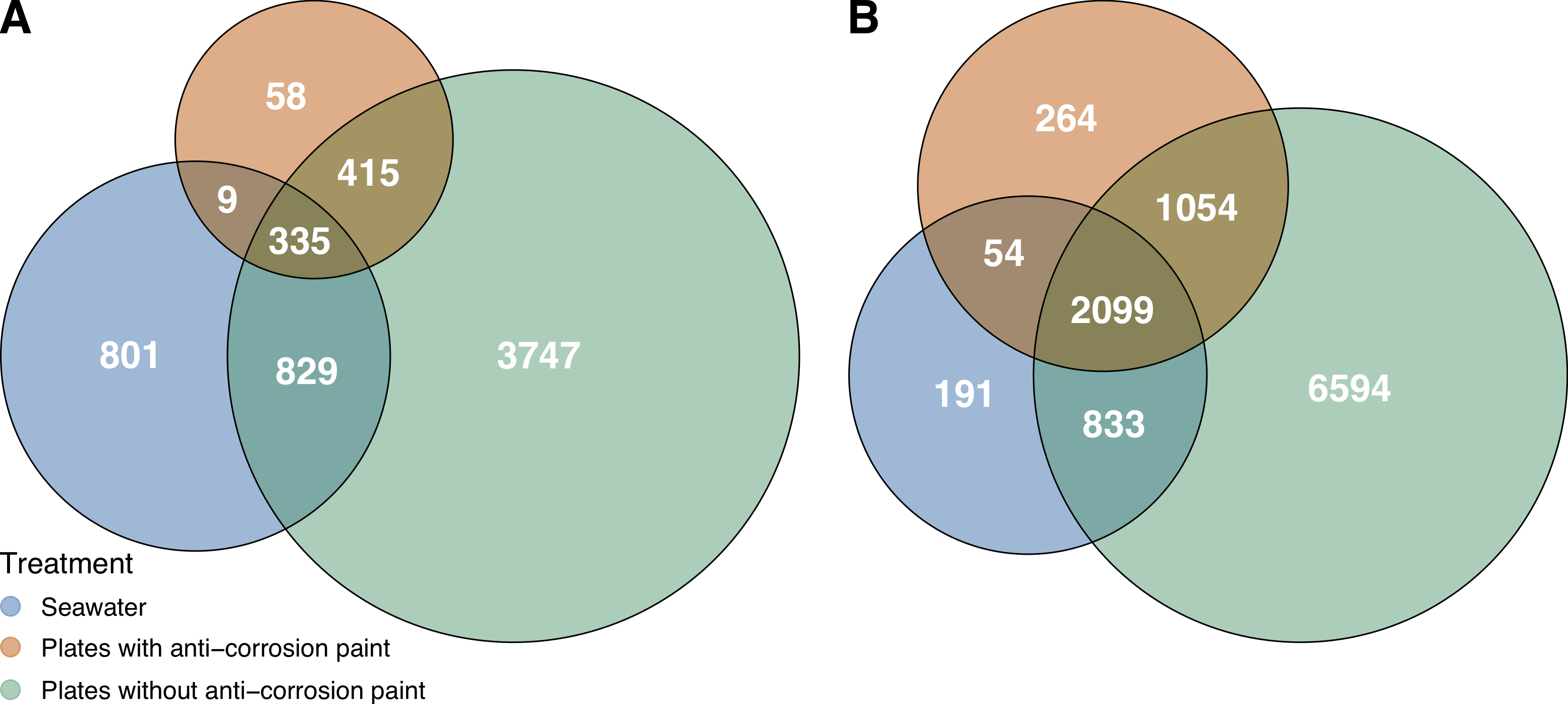

Supplement: FIGURE S4 — Venn diagram for the total OTUs for the different treatments for Spring (A) and for Winter (B). [file Image_4.tiff]

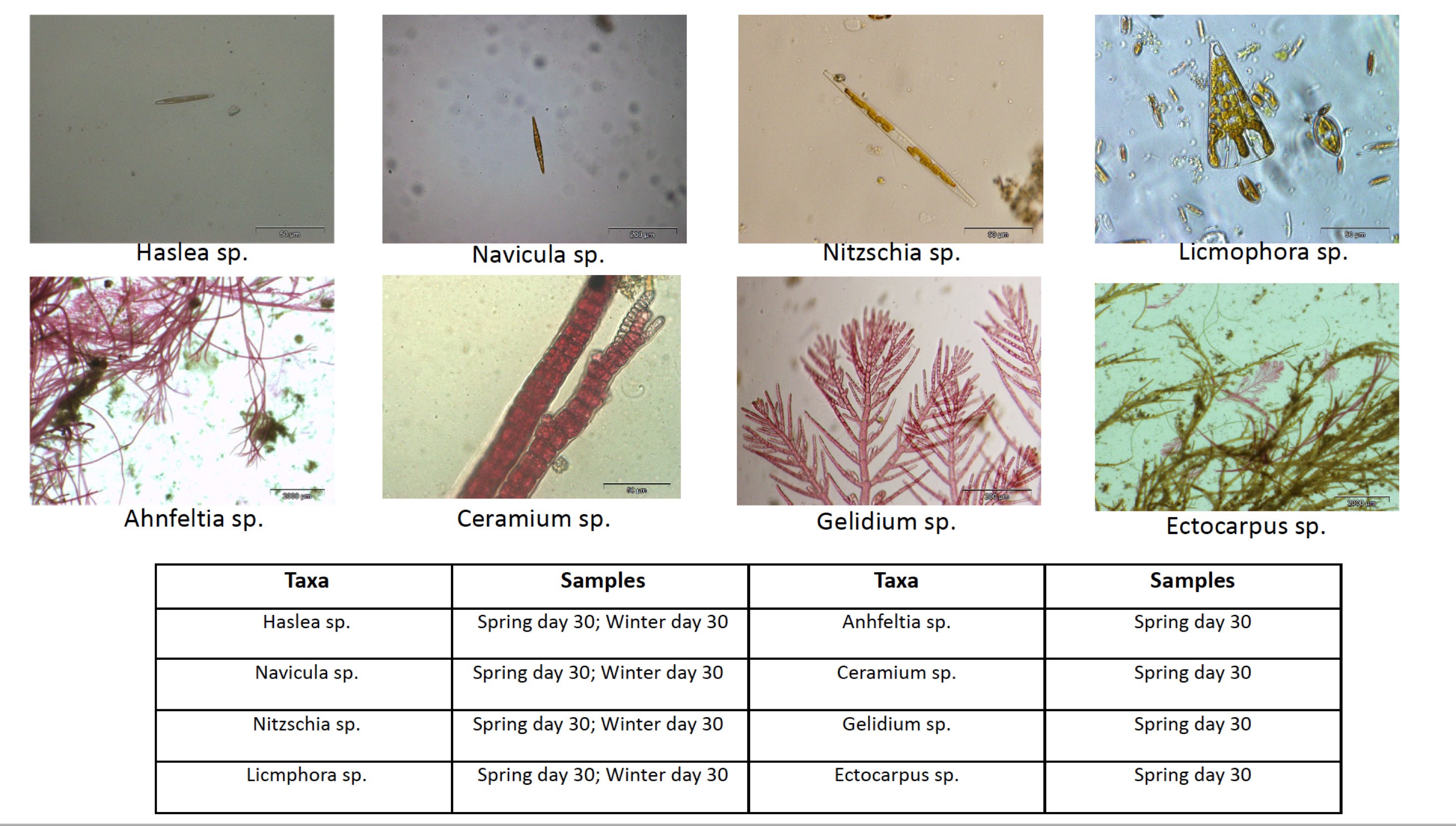

Supplement: FIGURE S5 — Macrofouling and phytoplankton species identified through microscopic observation in the last day of sampling of both seasons. [file Image_5.jpeg]
