# Supplementary material for: Distinct Temporal Succession of Bacterial Communities in Early Marine Biofilms in a Portuguese Atlantic Port
Source: Front Microbiol. 2020 Aug 11;11:1938. doi: 10.3389/fmicb.2020.01938 (PMC7432428; doi:10.3389/fmicb.2020.01938)
Supplement: TABLE S2 — Taxonomic abundance at the phylum level for the different treatments. [file Table_2.pdf]

Supplementary Table 2: Taxonomic abundance at the phylum level for the different treatments

2.1 Taxonomic abundance at the phylum level for the total samples

Taxa	Total
Acidobacteria	0.31 ± 0.07
Actinobacteria	7.75 ± 2.34
Bacteroidetes	15.6 ± 5.09
Cyanobacteria	14.9 ± 7.08
Firmicutes	0.43 ± 0.21
Planctomycetes	0.90 ± 0.45
Proteobacteria	52.2 ± 21.3
Thermi	1.47 ± 0.32
Tenericutes	0.27 ± 0.09
Other	1.13 ± 0.06

Values correspond to the average and standard error for each of the taxa. Phyla comprising <1% of the total number of sequences within a sample were simply classified as "Other".

2.2 Taxonomic abundance per season at the phylum level

Taxa	Spring	Winter
Acidobacteria	0.19 ± 0.17	0.39 ± 0.17
Actinobacteria	5.90 ± 4.48	12.8 ± 2.2
Bacteroidetes	11.4 ± 8.52	16.7 ± 12.1
Cyanobacteria	13.6 ± 10.4	9.97 ± 9.59
Firmicutes	0.71 ± 0.41	0.51 ± 0.43
Planctomycetes	1.46 ± 0.20	0.96 ± 0.19
Proteobacteria	51.8 ± 21.0	52.6 ± 19.7
Tenericutes	0.51±0.11	0.02 ± 0.01
Thermi	0.29 ± 0.15	2.88 ± 1.45
Other	2.49 ± 2.23	0.44 ± 0.37

Values correspond to the average and standard error for each of the taxa. Phyla comprising <1% of the total number of sequences within a sample were simply classified as "Other".

2.3 Taxonomic abundance per treatment at the phylum level

Taxa	Seawater	Plates without anticorrosion paint	Plates without anticorrosion paint
Acidobacteria	0.40 ± 0.30	0.38 ± 0.30	0.18 ± 0.07
Actinobacteria	11.32 ± 3.41	9.34 ± 3.19	4.93 ± 2.91
Bacteroidetes	18.36 ± 10.41	13.56 ± 10.51	17.1 ± 7.35
Cyanobacteria	4.62 ± 4.43	12.61 ± 11.32	8.03 ± 7.69
Firmicutes	0.41 ± 0.31	0.70 ± 0.62	0.23 ± 0.15
Planctomycetes	0.49 ± 0.40	0.42 ± 0.35	0.39 ± 0.34
Proteobacteria	57.98 ± 21.14	50.63 ± 19.53	60.77 ± 17.78
Thermi	1.35 ± 1.26	2.85 ± 1.47	2.83 ± 1.47
Tenericutes	0.00 ± 0.00	0.12 ± 0.02	0.00 ± 0.02
Other	0.99 ± 0.72	0.03 ± 0.02	0.82 ± 0.41

Values correspond to the average and standard error for each of the taxa. Phyla comprising <1% of the total number of sequences within a sample were simply classified as “Other”.

2.4 Taxonomic abundance per day for both seasons at the phylum level

Taxa	1	2	4	7	10	14	21	25	30
Acidobacteria	0.55± 0.45	0.90± 0.71	0.41± 0.34	0.61± 0.43	0.71± 0.34	1.97± 0.82	0.26± 0.31	0.31± 0.23	0.11± 0.08
Actinobacteria	10.12± 8.88	7.49± 5.88	13.41± 10.5	14.1± 5.57	13.9± 7.34	12.9± 5.09	4.56± 3.85	8.74± 6.42	4.33± 3.56
Bacteroidetes	5.67± 5.11	11.4± 10.6	4.78± 4.30	11.7± 4.63	10.4 ± 7.46	7.19± 3.21	24.1± 12.7	17.8± 7.72	17.4± 11.8
Cyanobacteria	7.23± 7.20	10.4± 7.74	13. 9± 3.73	10.3± 6.26	12.8± 7.31	7.20± 4.58	6.90± 5.61	7.61± 5.71	20.32± 19.4
Firmicutes	0.90± 0.51	1.31± 0.67	1.61± 0.54	0.71± 0.56	0.52± 0.45	2.07± 0.77	0.60± 0.56	1.16± 0.98	0.31± 0.27
Planctomycetes	1.19± 0.54	0.69± 0.54	0.59± 0.54	2.59± 0.32	1.79± 0.35	2.73± 2.31	1.45± 1.23	1.06± 0.67	0.76± 0.64
Proteobacteria	63.1± 6.11	55.2± 18.2	54.3± 24.4	44.7± 14.4	41.2± 21.9	57.9± 25.1	61.2± 11.7	57.3± 18.2	54.3± 23.8
Thermi	0.61± 0.54	0.61± 0. 54	0.82± 0.54	1.61± 0.54	5.61± 3.20	5.19± 3.20	0.55± 0.64	0.46± 0.37	0.45± 0.37
Tenericutes	0.21± 0.04	0.51± 0.04	0.12± 0.06	0.11± 0.03	0.10± 0.02	3.02E-05± 1.01E-05	1.46E-05± 1.00E-05	1.12E-05± 1.06E-05	1.47E-05± 1.03E-05
Other	0.82± 0.34	2.39± 1.05	0.50± 0.23	0.55± 0.18	2.95± 1.45	0.29± 0.15	0.17± 0.10	2.31± 1.07	1.98± 0.86

Values correspond to the average and standard error for each of the taxa. Phyla comprising <1% of the total number of sequences within a sample were simply classified as “Other”.

2.5 Taxonomic abundance at the phyla level per day during spring

Taxa	1	2	4	7	10	14	21	25	30	Ctr (30)	SW
Acidobacteria	0.31± 0.69	0.17± 0.69	1.50± 0.56	8.54± 3.34	0.05± 0.32	0.03± 0.62	0.003± 0.93	0.04± 1.07	0.04± 1.54	0.02± 1.54	0.59± 0.13
Actinobacteria	9.74± 4.88	13.4± 2.12	7.80± 3.99	12.2± 3.35	4.79± 2.61	2.93± 0.62	3.03± 0.92	3.03± 1.06	2.24± 10.3	0.02± 4.67	2.39± 1.24
Bacteroidetes	3.13± 5.9	2.67± 1.83	2.67± 6.22	7.97± 3.78	8.04± 9.55	10.5± 0.67	15.7± 2.20	15.7± 0.51	3.29± 4.75	17.3± 4.79	28.1± 14.2
Cyanobacteria	1.69± 0.69	5.82± 3.79	6.72± 6.26	7.32± 3.78	5.87± 1.12	6.89± 1.35	8.31± 6.68	8.30± 5.52	73.9± 4.04	8.18± 4.04	0.98± 2.01
Firmicutes	14.7± 5.76	11.8± 5.76	11.7± 3.21	7.37± 3.78	10.9± 4.67	0.81± 0.40	0.91± 0.93	0.61± 0.53	0.82± 0.41	1.61± 0.12	1.45± 0.33
Planctomycetes	1.54± 0.69	0.16± 2.51	0.25± 0.41	0.002± 0.45	1.62± 0.67	2.45± 0.67	0.30± 0.93	0.03± 1.43	0.04± 1.32	0.16± 0.12	0.21± 0.12
Proteobacteria	39.5± 18.1	34.3± 10.2	54.3± 14.4	41.9± 13.4	53.3± 19.6	68.4± 1.70	67.7± 3.99	67.7± 14.3	17.8± 23.1	54.9± 20.2	65.9± 23.2
Thermi	0.03± 0.69	1.04± 0.65	1.25± 0.75	4.09E-04± 0.01	7.32± 4.67	3.01E-04± 0.03	2.0E-04± 0.02	2.01E-04± 0.02	0.002± 1.2	0.06± 0.12	3.08E-05± 1.08E-0
Tenericutes	28.1± 0.69	22.6± 5.69	5.68± 2.69	3.79± 9.21	8.00± 4.67	8.01± 0.31	3.09± 0.93	3.09± 1.65	3.10± 2.31	7.16E-04± 0.02	0.09± 0.02
Other	1.15± 0.34	7.95 ±3.45	8.09 ±2.34	7.89 ±.2.43	0.07± 0.05	1.0E-04± 0.02	9.29± 4.32	1.25± 0.75	7.77± 3.23	1.25± 0.54	0.21± 0.12

Values correspond to the average and standard error for each of the taxa. Ctr (30) refers to plates with anti-corrosion paint; SW refers to seawater; Phyla comprising <1% of the total number of sequences within a sample were simply classified as “Other”.

2.6 Taxonomic abundance at the phyla level per day during winter

Taxa	1	2	4	7	10	14	21	25	30	Ctr (30)	SW
Acidobacteria	0.03± 0.74	0.004± 0.004	0.33± 0.16	0.50± 0.36	0.005± 0.003	0.004± 0.014	0.001± 0.014	0.62± 0.02	0.16± 0.05	0.007± 0.014	4.20E-04± 2.10E-04
Actinobacteria	8.84± 8.73	13.7± 10.4	0.43± 0.26	17.3± 8.31	17.8± 10.8	4.91± 5.51	6.03± 5.44	14.5± 13.5	7.17± 5.29	5.92± 3.43	8.92± 3.43
Bacteroidetes	11.5± 6.42	12.5± 4.30	13.3± 7.31	17.3± 4.37	17.8± 3.43	14.9± 5.18	20.5± 19.2	19.9± 11.7	21.5± 13.6	16.6± 2.35	12.0± 5.12
Cyanobacteria	23.3± 20.2	17.5± 18.9	18.3± 16.9	13.5± 7.18	21.0± 18.8	4.95± 3.71	4.03± 2.61	6.91± 7.76	4.49± 7.49	0.08± 0.04	11.8± 8.81

Firmicutes	0.84± 0.56	0.91± 0.63	1.03± 0.71	0.84± 0.62	0.04± 0.03	1.06± 0.07	2.38± 3.73	8.37± 2.66	1.73± 1.48	0.01± 0.03	0.05± 0.03
Planctomycetes	1.15± 0.64	0.004± 0.002	0.53± 0.45	1.36± 1.41	0.013± 0.009	0.014± 0.003	1.02± 0.78	3.34E-05 ± 1.34E-05	1.62± 0.75	0.39± 0.01	0.65± 0.21
Proteobacteria	51.6± 23.2	48.9± 18.5	59.5± 27.5	41.3± 7.67	36.3± 21.5	69.7± 28.7	64.2± 6.92	46.7± 21.9	61.3± 13.6	66.6± 23.3	63.4± 13.2
Thermi	0.008± 0.21	0.92± 0.57	0.93± 0.56	1.69± 1.41	0.054± 0.031	0.70± 0.012	4.61E-04± 2.12E-04	1.07± 1.41	2.01E-04± 2.01E-04	4.24E-04± 1.32E-04	1.85± 0.32
Tenericutes	4.08E-05± 1.23E-04	2.27E-05± 1.43E-04	1.47E-05± 5.67E-05	5.23E-05± 2.34E-05	1.16E-05± 5.67E-05	6.36E-05± 3.61E-05	5.26E-05± 3.62E-05	1.11E-05± 2.31E-05	1.47E-05± 1.21E-05	0.02± 0.01	4.47E-05± 2.23E-05
Other	2.67± 1.32	2.81± 1.94	5.53± 3.21	0.90± 0.34	3.20± 1.23	9.57± 4.56	3.75± 1.56	1.76± 0.56	1.97± 0.65	2.06± 1.31	1.59± 1.12

Values correspond to the average and standard error for each of the taxa. Ctr (30) refers to plates with anti-corrosion paint; SW refers to seawater; Phyla comprising <1% of the total number of sequences within a sample were simply classified as “Other”.
