# Supplementary material for: Distinct Temporal Succession of Bacterial Communities in Early Marine Biofilms in a Portuguese Atlantic Port
Source: Front Microbiol. 2020 Aug 11;11:1938. doi: 10.3389/fmicb.2020.01938 (PMC7432428; doi:10.3389/fmicb.2020.01938)
Supplement: TABLE S3 — Taxonomic abundance at the class level for the different treatments. [file Table_3.pdf]

Supplementary Table 3: Taxonomic abundance at the class level for the different treatments

3.1 Taxonomic abundance at the class level for the total samples

Taxa	Total
Actinobacteria	7.17 ± 2.46
Alphaproteobacteria	30.1 ± 0.65
Bacilli	0.20 ± 0.10
Betaproteobacteria	0.81 ± 0.50
Chloroplast	11.6 ± 3.69
Clostridia	0.33 ± 0.12
Cytopaghia	1.17 ± 0.94
Deltaproteobacteria	0.51 ± 0.23
Epsilonproteobacteria	2.04 ± 0.34
Flavobacteriia	14.1 ± 1.81
Gammaproteobacteria	25.0 ± 5.6
Mollicutes	0.03 ± 0.04
Oscillatoriophycideae	2.87 ± 1.93
Planctomycetia	0.37 ± 0.17
Saprospirae	1.43 ± 0.21
Other	1.65 ± 0.32

Values correspond to the average and standard error for each of the taxa. Classes comprising <1% of the total number of sequences within a sample were simply classified as "Other".

3.2 Taxonomic abundance per season at the class level

Taxa	Spring	Winter
Actinobacteria	1.93 ± 1.53	5.65 ± 2.21
Alphaproteobacteria	33.1 ± 11.8	25.6 ± 12.9
Bacilli	0.21 ± 0.52	0.20 ± 0.60
Betaproteobacteria	1.26 ± 1.52	1.00 ± 0.60
Chloroplast	14.4 ± 3.51	4.32 ± 3.61
Clostridia	1.66 ± 0.34	0.71 ± 1.28
Cytopaghia	1.71 ± 0.53	0.65 ± 0.33
Deltaproteobacteria	0.52 ± 0.43	0.50 ± 0.34
Epsilonproteobacteria	1.67 ± 1.22	2.95 ± 2.41
Flavobacteriia	14.1 ± 13.2	12.9 ± 10.8
Gammaproteobacteria	21.3 ± 3.81	33.9 ± 22.4
Mollicutes	0.51 ± 0.31	0.01 ± 0.24
Oscillatoriophycideae	2.12 ± 0.51	0.94 ± 0.74

Planctomycetia	1.11 ± 0.30	0.50 ± 0.21
Saprospirare	1.11 ± 0.32	1.80 ± 0.12
Other	1.78 ± 0.87	1.32 ± 0.23

Values correspond to the average and standard error for each of the taxa. Classes comprising <1% of the total number of sequences within a sample were simply classified as "Other".

3.3 Taxonomic abundance per treatment at the class level

**Taxa**	**Seawater**	**Plates without anticorrosion paint**	**Plates with anticorrosion paint**
Actinobacteria	10.6 ± 3.28	8.9 ± 3.7	3.6 ± 3.2
Alphaproteobacteria	30.8 ± 13.8	28.9 ± 15.7	29.4 ± 9.81
Bacilli	0.10 ± 0.08	3.14 ± 1.85	0.36± 0.31
Betaproteobacteria	0.50 ± 0.42	1.57 ± 1.26	0.36 ± 0.31
Chloroplast	6.39 ± 4.68	14.5 ± 7.58	13.5 ± 7.59
Clostridia	0.36 ± 0.28	0.56 ± 0.15	0.94 ± 0.51
Cytopaghia	0.85 ± 0.72	2.59 ± 0.68	0.47 ± 0.22
Deltaproteobacteria	0.30 ± 0.24	0.27 ± 0.12	0.27 ± 0.12
Epsilonproteobacteria	0.49 ± 0.41	2.31 ± 0.78	0.57 ± 0.38
Flavobacteriia	13.6 ± 12.0	13.1 ± 8.76	16.7 ± 2.93
Gammaproteobacteria	24.8 ± 15.4	15.3 ± 11.1	27.5 ± 13.6
Mollicutes	0.10 ± 0.04	0.08± 0.27	0.01 ± 0.08
Oscillatoriophycideae	5.42 ± 3.46	2.59 ± 2.41	0.71 ± 0.62
Planctomycetia	0.21 ± 0.04	0.66 ± 0.34	0.31 ± 0.24
Saprospirae	1.71 ± 1.61	1.78 ± 1.41	1.46 ± 0.99
Other	2.01 ± 1.03	1.45 ± 0.56	1.04 ± 0.34

Values correspond to the average and standard error for each of the taxa. Classes comprising <1% of the total number of sequences within a sample were simply classified as "Other".

3.4 Taxonomic abundance per day at the class level

**Taxa**	**1**	**2**	**4**	**7**	**10**	**14**	**21**	**25**	**30**
Actinobacteria	6.52± 6.04	21.3± 6.5	5.41± 2.77	13.0 ± 8.45	10.2± 7.21	14.6± 11.1	3.51± 4.24	7.51± 6.42	3.12± 3.08
Alphaproteobacteria	28.3± 13.5	30.6± 14.4	36.4± 14.1	31.0± 9.88	25.9± 14.5	32.6± 21.6	36.6± 12.3	36.1± 13.2	21.6± 9.17
Bacilli	0.26± 0.05	0.43± 0.13	0.25± 0.80	0.31± 0.24	0.11± 0.04	0.21± 0.10	0.11± 0.12	0.41± 0.12	0.41± 0.12
Betaproteobacteria	1.31± 0.24	3.64± 1.49	2.41± 0.83	1.70± 0.45	1.25± 0.01	1.21± 0.47	1.42± 0.83	0.66± 0.28	3.43± 2.46

Chloroplast	20.0± 16.1	5.94± 7.48	16.1± 5.83	9.31± 7.87	18.6± 13.3	3.21± 0.47	11.3± 10.6	4.84± 0.69	13.7± 6.78
Clostridia	12.1± 4.50	0.93± 0.49	0.75± 0.18	0.61± 0.21	0.31± 0.12	0.32± 0.17	0.53± 0.23	0.71± 0.23	0.21± 0.45
Cytopaghia	0.92± 0.61	3.94± 1.65	1.15± 0.83	2.10± 1.27	6.31± 2.34	7.23± 4.34	0.61± 0.49	0.91± 0.39	0.41± 0.26
Deltaproteobacteria	0.86± 0.61	0.34± 0.19	0.32± 0.18	1.10± 0.77	0.61± 0.34	0.81± 0.24	0.51± 0.13	0.81± 0.29	0.31± 0.21
Epsilonproteobacteria	2.23± 0.61	1.44± 0.68	4.75± 0.83	2.24± 0.83	1.11± 0.56	2.11± 0.34	1.77± 1.45	1.32± 0.25	1.67± 1.22
Flavobacteriia	8.15± 6.00	6.12± 3.78	9.26± 4.15	6.10± 4.27	7.81± 5.65	7.74± 3.14	14.8± 9.7	13.8± 8.21	22.1± 15.3
Gammaproteobacteria	10.6± 7.93	8.65± 7.67	15.4± 9.81	13.1± 6.50	11.9± 5.71	11.8± 5.67	17.3± 9.80	21.4± 8.21	36.9± 21.1
Mollicutes	0.16± 0.06	0.55± 0.18	0.14± 0.08	0.11± 0.08	0.11± 0.05	0.10± 0.35	0.06± 0.04	0.04± 0.05	0.03± 0.02
Oscillatoriophycideae	1.36± 0.74	8.11± 3.90	1.61± 1.24	5.90± 2.88	2.23± 1.93	3.61± 2.20	4.07± 1.79	4.02± 3.17	0.54± 0.41
Planctomycetia	0.39± 0.29	0.22± 0.10	0.32± 0.25	0.90± 0.08	0.61± 0.05	0.81± 0.03	1.90± 1.57	1.71± 0.47	0.05± 0.03
Saprospirae	1.79± 0.59	1.52± 0.77	1.52± 0.77	1.04± 0.61	2.32± 0.56	1.76± 0.56	1.96± 0.41	1.72± 0.90	1.23± 0.76
Other	2.03± 1.23	1.98± 0.76	3.41± 0.45	0.98± 0.43	1.21± 0.32	2.06± 3.12	2.45± 0.85	1.23± 0.95	1.31± 0.78

Values correspond to the average and standard error for each of the taxa. Classes comprising <1% of the total number of sequences within a sample were simply classified as "Other".

3.5 Taxonomic abundance at the class level per day during spring

**Taxa**	**1**	**2**	**4**	**7**	**10**	**14**	**21**	**25**	**30**	**Ctr (30)**	**SW**
Actinobacteria	9.57± 4.93	13.1± 3.22	7.56± 1.09	11.8± 6.52	3.93± 3.0	1.52± 0.64	1.56± 1.14	2.01± 0.34	2.23± 2.05	1.64± 0.05	1.00± 0.33
Alphaproteobacteria	28.6± 5.89	14.6± 7.85	16.2± 9.52	21.1± 15.2	31.9± 7.11	59.6± 2.77	36.3± 3.97	38.6± 6.16	12.3± 9.09	22.3± 9.09	38.3± 15.6
Bacilli	2.65± 1.24	5.12± 1.62	1.44± 1.33	3.26± 2.42	2.62± 0.30	0.20± 0.11	0.03± 0.21	0.04± 0.16	0.06± 0.01	0.82± 0.01	0.17± 0.06
Betaproteobacteria	3.77± 1.44	4.50± 1.74	1.53± 0.78	4.21± 1.34	2.62± 0.31	1.97± 0.36	1.47± 0.36	1.31± 0.16	0.37± 0.14	0.40± 0.14	0.80± 0.23
Chloroplast	0.74± 0.34	1.19± 0.30	0.98± 0.71	0.76± 0.48	3.55± 0.47	0.01± 0.006	19.8± 9.82	6.31± 1.28	73.1± 1.87	7.60± 1.67	0.86± 0.16
Clostridia	12.1± 1.96	14.3± 3.56	2.70± 1.31	7.10± 1.32	9.63± 2.74	0.61± 0.23	0.20± 0.34	0.15± 0.10	0.15± 0.07	4.93± 0.07	1.22± 0.31
Cytopaghia	0.73±	0.69±	3.71±	1.83±	0.67±	0.79±	0.65±	1.40±	0.37±	0.34±	0.05±

	0.51	0.48	1.45	1.66	0.52	0.34	0.23	0.34	0.07	0.07	0.02
Deltaproteobacteria	0.70± 0.51	0.99± 0.67	0.18± 0.03	0.31± 0.02	0.99± 0.31	0.51± 0.22	0.24± 0.12	0.56± 0.32	0.63± 0.41	0.23± 0.08	0.15± 0.06
Epsilonproteobacteria	4.12± 2.31	1.30± 0.56	3.05± 1.43	3.30± 1.23	1.82± 0.64	0.62± 0.31	1.30± 0.62	0.55± 0.11	0.27± 0.34	0.05± 0.21	1.56± 0.56
Flavobacteriia	20.6± 7.54	1.28± 1.12	2.83± 1.45	3.95± 2.28	8.81± 3.41	0.62± 0.22	11.4± 2.46	11.4± 0.78	2.21± 1.34	17.3± 1.34	26.9± 7.74
Gammaproteobacteria	20.8± 5.67	13.2± 2.40	43.7± 13.4	19.7± 8.65	15.9± 3.01	11.2± 0.93	11.7± 1.02	26.3± 5.11	4.02± 2.96	31.3± 3.96	25.8± 8.72
Mollicutes	2.87± 1.23	22.2± 12.2	3.68± 2.71	1.31± 0.78	1.64± 0.56	0.10± 0.93	0.01± 0.03	0.09± 0.04	0.03± 0.05	1.09± 3.96	0.006± 0.002
Oscillatoriophycideae	2.83± 1.24	3.89± 2.21	7.57± 4.56	6.11± 4.86	1.19± 0.13	3.82± 1.23	3.06± 0.55	1.84± 0.11	1.52± 0.36	0.32± 0.26	0.008± 0.003
Planctomycetia	0.14± 0.05	0.12± 0.07	0.12± 0.05	0.43± 0.22	1.62± 0.12	1.40± 1.19	2.67± 0.69	1.76± 0.11	1.35± 0.45	0.20± 0.05	0.21± 0.06
Saprospirae	0.24± 0.14	0.33± 0.15	0.13± 0.03	1.04± 0.56	3.09± 1.24	1.02± 0.65	2.19± 0.19	2.00± 0.31	0.42± 0.13	0.06± 0.03	0.20± 0.07
Other	2.56± 1.89	2.43± 0.78	1.78± 0.45	2.67± 1.35	2.41± 0.87	3.01± 2.87	2.67± 0.87	1.56± 0.76	1.45± 0.87	1.76± 0.89	3.06± 1.02

Values correspond to the average and standard error for each of the taxa. Ctr (30) refers to plates with anti-corrosion paint; SW refers to seawater; Classes comprising <1% of the total number of sequences within a sample were simply classified as “Other”.

3.6 Taxonomic abundance at the class level per day during winter

Taxa	1	2	4	7	10	14	21	25	30	Ctr	SW
Actinobacteria	8.30± 6.22	12.5± 7.72	5.67± 2.32	15.6± 2.36	7.20± 5.47	4.41± 0.33	5.74± 3.80	6.37± 0.03	5.56± 3.67	5.49± 1.49	20.1± 9.31
Alphaproteobacteria	33.8± 14.0	34.9± 5.32	32.2± 17.8	29.8± 4.51	32.2± 11.9	44.7± 19.5	37.8± 12.1	28.0± 13.5	31.8± 18.7	21.8± 11.8	18.0± 5.61
Bacilli	0.40± 0.29	1.41± 0.97	0.20± 0.34	0.37± 1.86	0.09± 0.90	0.27± 0.32	0.47± 0.75	0.43± 0.36	0.50± 0.20	0.30± 0.11	1.59± 0.23
Betaproteobacteria	1.59± 1.29	2.59± 1.97	1.98± 1.61	2.31± 1.86	1.58± 0.90	1.19± 0.32	1.20± 0.76	1.04± 0.47	1.09± 0.50	0.36± 0.15	1.49± 0.35
Chloroplast	13.1± 4.56	13.1± 6.99	10.0± 9.30	7.85± 6.82	20.6± 14.9	1.19± 0.46	3.12± 2.56	2.01± 1.17	2.66± 1.43	7.30± 4.30	4.52± 1.24
Clostridia	0.44± 0.29	2.33± 1.98	0.70± 0.01	0.37± 0.74	0.25± 0.16	0.60± 0.23	1.91± 0.49	4.76± 1.26	1.22± 0.10	0.94± 0.44	5.85± 3.21
Cytophagia	1.10± 0.56	2.10± 1.98	1.53± 1.00	2.36± 1.75	1.27± 1.16	6.75± 3.23	0.61± 0.19	0.93± 0.21	0.64± 0.34	0.57± 0.32	0.49± 0.21
Deltaproteobacteria	0.74± 0.55	2.79± 0.79	0.86± 0.66	0.79± 0.52	0.58± 0.31	3.18± 2.76	0.51± 0.64	0.45± 0.27	0.49± 0.34	0.12± 0.09	0.31± 0.12
Epsilonproteobacteria	1.74± 0.87	0.90± 0.68	2.64± 0.55	2.23± 0.13	1.63± 0.45	4.04± 2.21	2.35± 0.34	1.42± 0.71	3.50± 2.50	0.52± 0.13	0.11± 0.05
Flavobacteriia	7.82±	7.66±	9.30±	8.45±	10.8±	10.4±	18.5±	23.1±	19.5±	14.6±	6.37±

	0.67	2.23	4.85	4.91	2.01	3.05	10.3	9.50	4.05	7.21	1.21
Gammaproteobacteria	12.6± 7.47	10.1± 0.27	8.67± 2.34	9.73± 0.99	12.5± 2.11	18.8± 4.49	22.5± 3.75	28.0± 7.56	24.3± 6.78	43.7± 12.4	29.9± 3.32
Mollicutes	0.01± 0.02	0.06± 0.02	0.05± 0.06	0.008± 0.005	0.001± 0..003	0.006± 0.005	0.01± 0.01	0.01± 0.04	0.001± 0.005	0.02± 0.01	0.03± 0.02
Oscillatoriophycideae	1.40± 0.71	3.99± 1.56	4.51± 0.87	7.80± 5.99	1.68± 1.59	0.83± 0.17	0.88± 0.38	0.55± 0.27	1.50± 0.67	0.71± 0.19	3.73± 1.34
Planctomycetia	1.25± 0.44	0.55± 0.32	1.31± 1.29	1.37± 0.72	1.36± 0.82	1.42± 0.54	0.61± 0.21	0.34± 0.21	2.31± 1.11	0.39± 0.16	0.009± 0.005
Saprospirae	1.78± 1.03	2.07± 1.54	2.76± 0.45	3.02± 2.72	2.89± 145	2.43± 1.42	2.08± 0.67	1.36± 0.43	0.76± 0.56	0.57± 0.12	1.41± 0.34
Other	2.31 ±1.09	3.45± 1.31	2.31± 0.98	3.01± 2.08	2.16± 1.51	2.61± 0.98	3.90± 2.10	3.71± 2.09	1.07± 0.34	2.03± 0.93	2.89± 9.98

Values correspond to the average and standard error for each of the taxa. Ctr (30) refers to plates with anti-corrosion paint; SW refers to seawater; Classes comprising <1% of the total number of sequences within a sample were simply classified as “Other”.
