# Supplementary material for: Distinct Temporal Succession of Bacterial Communities in Early Marine Biofilms in a Portuguese Atlantic Port
Source: Front Microbiol. 2020 Aug 11;11:1938. doi: 10.3389/fmicb.2020.01938 (PMC7432428; doi:10.3389/fmicb.2020.01938)
Supplement: TABLE S4 — Taxonomic abundance at the order level for the different treatments. [file Table_4.pdf]

Supplementary Table 4: Taxonomic abundance at the order level for the different treatments

4.1 Taxonomic abundance at the class level for the total samples

Taxa	Total
Actinomycetales	7.79 ± 2.63
Alteromonadales	5.23 ± 3.02
Bacteroidales	0.54 ± 0.12
Burkholderiales	7.81 ± 3.02
Campylobacterales	2.28 ± 1.05
Chroococcales	6.40 ± 2.09
Clostridiales	2.20 ± 0.65
Cytophagales	1.46 ± 0.34
Deinococcales	0.76 ± 0.21
Flavobacteriales	8.73 ± 4.02
Methylococcales	0.31 ± 0.12
Mycoplasmatales	9.28 ± 2.80
Oceanospirillales	6.48 ± 2.14
Oscillatoriales	1.58 ± 0.56
Pseudomonadales	2.03 ± 0.65
Rhizobiales	3.36 ± 0.32
Rhodobacterales	15.7 ± 4.60
Rhodospirillales	1.51 ± 0.73
Saprospirales	2.73 ± 1.23
Sphingobacteriales	2.31 ± 0.65
Sphingomonodales	2.71 ± 1.09
Stramenopiles	11.1 ± 4.31
Vibrionales	2.72 ± 0.87
Other	4.31 ± 4.09

Values correspond to the average and standard error for each of the taxa. Orders comprising <1% of the total number of sequences within a sample were simply classified as “Other”.

4.2 Taxonomic abundance per season at the class level

Taxa	Spring	Winter
Actinomycetales	5.79 ± 4.80	11.4 ± 6.78
Alteromonadales	6.24 ± 3.45	4.92 ± 3.08
Bacteroidales	0.84 ± 0.88	0.37 ± 0.02
Burkholderiales	10.8 ± 6.79	1.18 ± 1.02
Campylobacterales	2.29 ± 1.94	2.09 ± 1.80

Chroococcales	2.66 ± 1.32	1.41 ± 2.14
Clostridiales	6.40 ± 3.42	0.41 ± 0.14
Cytophagales	1.20 ± 0.56	3.55 ± 2.16
Deinococcales	0.20 ± 0.12	1.94 ± 0.75
Flavobacteriales	14.2 ± 8.60	11.6 ± .7.15
Methylococcales	4.29 ± 0.59	1.23 ± 0.98
Mycoplasmatales	0.23± 0.08	0.001± 0.005
Oceanospirillales	6.48 ± 5.67	2.26 ± 1.82
Oscillatoriales	1.58 ± 1.30	2.56 ± 1.25
Pseudomonadales	2.03 ± 1.91	1.18 ± 0.84
Rhizobiales	3.36 ± 2.28	5.14 ± 2.61
Rhodobacterales	24.7 ± 14.3	19.4 ± 12.9
Rhodospirillales	1.51 ± 1.03	1.12 ± 0.96
Saprospirales	1.21 ± 0.08	1.40 ± 1.61
Sphingobacteriales	3.23 ± 2.72	1.54 ± 0.83
Sphingomonodales	3.72 ± 2.72	3.36 ± 2.27
Stramenopiles	13.2 ± 1.34	7.12 ± 13.3
Vibrionales	3.21 ± 1.45	2.31 ± 1.89
Other	2.34 ± 0.89	3.31 ± 0.32

Values correspond to the average and standard error for each of the taxa. Classes comprising <1% of the total number of sequences within a sample were simply classified as “Other”.

4.3 Taxonomic abundance per treatment at the class level

Taxa	Seawater	Plates without anticorrosion paint	Plates with anticorrosion paint
Actinomycetales	9.82 ± 7.16	8.66 ± 5.59	3.83 ± 1.23
Alteromonadales	12.4 ± 5.43	3.65 ± 2.71	8.22 ± 4.24
Bacteroidales	0.78 ± 0.54	0.45 ± 0.31	0.16 ± 0.03
Burkholderiales	0.47 ± 0.21	1.05 ± 0.70	1.00 ± 0.90
Campylobacterales	1.56 ± 0.56	2.45 ± 0.70	2.39 ± 1.06
Chroococcales	1.41 ± 1.14	0.90 ± 0.31	0.31 ± 0.09
Clostridiales	1.21 ± 0.93	0.45 ± 0.20	0.21 ± 0.03
Cytophagales	0.05 ± 0.02	2.45 ± 0.61	0.63 ± 0.38
Deinococcales	2.18 ± 1.51	1.95 ± 0.20	0.52 ± 0.08
Flavobacteriales	1.84 ± 0.76	11.9 ± 7.06	17.8 ± 6.54
Methylococcales	0.02 ± 0.01	2.19 ± 0.70	3.63 ± 1.04
Mycoplasmatales	0.03 ± 0.01	0.19 ± 0.07	0.03 ± 0.04
Oceanospirillales	7.50 ± 1.56	2.19 ± 3.41	11.3 ± 4.34
Oscillatoriales	3.49 ± 1.57	1.59 ± 0.78	2.02 ± 1.29
Pseudomonadales	0.66 ± 0.31	1.19 ± 0.65	1.65 ± 1.15
Rhizobiales	2.27 ± 1.24	2.41 ± 1.22	0.95 ± 0.62
Rhodobacterales	24.3 ± 15.9	22.1 ± 9.96	19.6 ± 12.7

Rhodospirillales	1.10 ± 0.61	1.09 ± 0.95	0.88 ± 1.14
Saprospirales	1.40 ± 0.41	1.41 ± 0.56	2.30 ± 1.61
Sphingobacteriales	0.84 ± 0.34	0.21 ± 0.13	0.21 ± 0.13
Sphingomonodales	1.84 ± 1.39	3.01 ± 1.61	2.71 ± 1.78
Stramenopiles	6.19 ± 5.67	14.2 ± 7.73	13.2 ±1.96
Vibrionales	2.00 ± 1.31	2.24 ± 0.21	3.30 ± 1.60
Other	3.31 ± 1.09	2.71 ± 0.65	3.02 ± 0.56

Values correspond to the average and standard error for each of the taxa. Classes comprising <1% of the total number of sequences within a sample were simply classified as "Other".

4.4 Taxonomic abundance per day for both seasons at the order level

**Taxa**	**1**	**2**	**4**	**7**	**10**	**14**	**21**	**25**	**30**
Actinomycetales	6.58± 3.32	21.8± 6.96	5.38± 3.03	12.7± 7.85	14.5± 4.56	13.5± 6.31	3.30± 2.72	5.10± 3.80	3.56± 2.05
Alteromonadales	3.07± 2.34	1.75± 1.55	2.28± 0.98	3.74± 1.36	2.86± 1.40	4.46± 2.89	5.91± 3.81	5.91± 2.65	9.41± 8.13
Bacteroidales	0.50± 0.23	0.49± 0.16	0.51± 0.09	0.90± 0.52	0.36± 0.21	0.20± 0.12	0.31± 0.18	0.51± 0.33	0.17± 0.10
Burkholderiales	0.88± 0.46	3.32± 2.78	1.42± 1.02	1.34± 0.36	0.70± 0.60	0.70± 0.50	0.52± 0.16	1.39± 1.16	0.22± 0.50
Campylobacterales	2.25± 0.50	1.49± 1.16	4.77± 0.64	2.84± 1.56	1.19± 0.23	2.31± 1.46	2.77± 1.45	1.27± 0.29	0.86± 0.71
Chroococcales	0.51± 0.12	3.10± 0.41	1.16± 0.79	1.16± 0.19	0.74± 0.51	0.46± 0.19	0.72± 0.41	0.58± 0.29	0.03± 0.05
Clostridiales	0.62± 0.12	0.92± 0.12	0.69± 0.37	0.66± 0.15	0.31± 0.12	1.36± 0.45	0.19± 0.01	1.25± 0.13	0.26± 0.15
Cytophagales	0.84± 0.19	3.89± 2.16	1.20± 0.82	2.66± 1.02	6.47± 2.34	7.74± 2.12	0.52± 0.33	0.90± 0.73	0.40± 0.10
Deinococcales	0.74± 0.19	2.79± 1.16	0.90± 0.22	1.69± 0.62	5.47± 3.32	4.26± 1.26	0.43± 0.34	0.66± 0.52	0.42± 0.19
Flavobacteriales	7.41± 3.12	6.48± 2.69	9.20± 4.44	9.93± 4.58	8.40± 3.41	7.71± 5.12	21.8± 13.6	13.8± 9.00	19.3± 12.4
Methylococcales	0.13± 0.03	0.13± 0.03	0.01± 0.03	0.43± 0.17	1.20± 0.23	0.17± 0.06	8.13± 3.63	0.62± 0.43	5.41± 1.56
Mycoplasmatales	0.23± 0.03	0.51± 0.03	0.05± 0.03	0.13± 0.08	0.10± 0.04	0.05± 0.01	0.31± 0.05	0.01± 0.43	0.02± 0.03
Oceanospirillales	2.85± 1.56	2.32± 0.39	2.76± 1.21	1.22± 0.34	1.56± 0.74	1.70± 1.55	2.24± 1.70	3.62± 1.92	11.7± 7.8
Oscillatoriales	1.06± 0.37	4.67± 0.91	0.52± 0.15	3.80± 2.26	2.62± 1.21	2.09± 1.73	0.61± 0.44	3.05± 1.74	0.40± 0.19
Pseudomonadales	1.13± 0.43	1.77± 0.91	1.96± 0.80	2.55± 1.01	1.15± 0.34	0.96± 0.45	0.71± 0.24	1.42± 1.01	0.14± 0.02
Rhizobiales	2.45± 0.89	4.20± 1.57	1.92± 0.46	2.01± 0.75	1.90± 0.45	2.73± 1.32	3.98± 0.84	1.81± 0.43	0.91± 0.45
Rhodobacterales	22.0±	15.6±	28.2±	21.0±	21.0±	20.7±	23.9±	30.5±	21.2±

	15.7	8.61	10.6	10.0	15.1	7.89	10.4	12.5	10.3
Rhodospirillales	1.20± 0.96	2.73± 1.11	1.53± 1.23	1.77± 0.90	1.15± 0.77	1.32± 0.56	0.47± 0.23	1.24± 1.14	0.34± 0.21
Saprospirales	1.45± 0.76	1.53± 1.11	1.86± 0.45	3.04± 0.43	2.32± 1.01	1.72± 0.43	1.10± 0.23	2.27± 0.97	1.03± 0.86
Sphingobacteriales	1.72± 0.56	0.67± 0.34	0.10± 0.06	0.41± 0.19	2.02± 1.21	0.23± 0.19	0.11± 0.03	5.86± 5.31	0.13± 0.06
Sphingomonodales	1.82± 1.34	6.73± 0.84	3.50± 2.21	4.46± 2.12	2.73± 0.41	4.14± 0.27	1.26± 0.02	2.40± 1.27	2.31± 1.47
Stramenopiles	21.8± 15.5	5.72± 2.21	16.3± 9.45	9.09± 6.43	18.3± 15.1	3.23± 2.55	4.67± 2.08	3.70± 1.45	13.6± 11.4
Vibrionales	0.62± 0.46	1.90± 0.64	4.00± 1.65	0.54± 0.18	0.74± 0.21	0.43± 0.18	4.97± 2.08	7.58± 5.22	2.75± 0.70
Other	2.41± 1.45	4.35± 2.01	1.87± 0.67	5.31± 2.56	4.31± 2.14	3.21± 0.98	3.19± 0.97	3.01± 1.06	1.05± 0.43

Values correspond to the average and standard error for each of the taxa. Classes comprising <1% of the total number of sequences within a sample were simply classified as "Other".

4.5 Taxonomic abundance at the class level per day during spring

**Taxa**	**1**	**2**	**4**	**7**	**10**	**14**	**21**	**25**	**30**	**Ctr (30)**	**SW**
Actinomycetales	9.57± 4.93	13.1± 2.31	7.60± 3.90	10.1 ± 3.67	10.1± 2.63	3.87± 1.25	2.61± 0.93	2.04± 0.98	0.88± 0.69	1.63± 0.43	0.87± 0.51
Alteromonadales	0.16± 0.12	0.57± 0.44	0.97± 0.24	5.81± 1.46	2.96± 0.45	8.08± 0.02	7.72± 0.56	5.92± 0.33	3.96± 1.45	1.65± 0.41	16.9± 7.65
Bacteroidales	1.15± 0.45	0.07± 0.03	0.10± 0.06	0.90± 0.52	0.87± 0.34	0.63± 0.05	0.17± 0.06	0.09± 0.05	0.06± 0.03	0.07± 0.04	0.02± 0.05
Burkholderiales	3.47± 2.63	4.65± 3.65	1.22± 1.02	9.35± 2.65	9.34± 2.11	17.2± 5.64	4.05± 2.31	6.51± 0.45	3.31± 2.18	0.02± 0.06	3.32± 2.01
Campylobacterales	0.70± 0.24	0.57± 0.31	3.38± 0.64	3.45± 2.31	3.45± 1.43	2.01± 0.56	1.20± 0.56	2.32± 0.76	1.82± 0.55	1.64± 0.78	0.12± 0.05
Chroococcales	0.51± 0.12	1.84± 0.75	0.52± 0.23	5.57± 2.86	0.56± 0.31	0.21± 0.09	2.22± 1.40	1.23± 0.56	1.02± 0.56	0.98± 0.45	0.08± 0.12
Clostridiales	12.1± 3.43	14.3± 2.53	2.07± 1.67	5.29± 2.26	7.21± 2.19	6.23± 0.56	0.20± 0.09	0.15± 0.08	0.36± 0.21	0.43± 0.31	0.41± 0.06
Cytophagales	0.05± 0.02	0.70± 0.56	2.70± 1.82	1.66± 0.37	1.66± 0.85	0.77± 0.23	0.58± 0.20	1.39± 0.37	1.56± 0.21	0.31± 0.21	0.31± 0.05
Deinococcales	0.01± 0.04	1.41± 0.12	1.53± 0.05	1.54± 0.76	1.65± 0.45	0.95± 0.23	0.81± 0.65	0.22± 0.12	0.12± 0.05	0.41± 0.06	1.92± 0.65
Flavobacteriales	1.09± 0.53	1.29± 1.01	2.83± 1.77	6.46± 2.18	5.45± 2.86	7.65± 2.34	10.2± 1.81	11.4± 2.35	2.35± 0.41	17.2± 9.07	27.3± 6.54
Methylococcales	0.20± 0.06	0.01± 0.04	0.03± 0.02	0.04± 0.21	0.01± 0.05	0.03± 0.04	1.21± 0.56	1.10± 0.80	0.67± 0.43	2.19± 0.56	3.63± 0.56
Mycoplasmatales	28.2± 9.34	22.7± 6.43	0.68± 2.85	0.96± 3.31	0.56± 0.43	0.02± 0.01	0.01± 0.01	0.01± 0.06	0.02± 0.05	0.01± 0.04	0.00± 0.11

Oceanospirillales	18.8± 5.40	9.02± 7.05	6.61± 2.60	2.46± 1.51	2.27± 1.21	1.92± 0.56	0.64± 0.32	3.50± 2.21	0.13± 0.05	0.30± 0.05	6.61± 2.45
Oscillatoriales	0.21± 0.15	2.05± 0.96	1.52± 1.15	1.38± 1.02	0.44± 0.10	2.31± 0.26	1.82± 0.76	1.64± 0.67	1.32± 0.78	0.21± 0.06	0.10± 0.05
Pseudomonadales	2.28± 0.24	2.42± 0.88	4.72± 1.62	3.99± 2.99	1.84± 1.03	0.14± 0.09	0.25± 0.30	0.50± 0.23	0.05± 0.12	0.61± 0.23	0.68± 0.32
Rhizobiales	2.66± 0.36	2.99± 1.84	3.29± 1.73	1.79± 0.25	3.51± 1.68	0.23± 0.10	1.75± 0.37	1.81± 0.43	2.73± 1.21	1.06± 0.34	0.12± 0.70
Rhodobacterales	1.50± 0.78	1.43± 0.17	4.81± 0.77	18.5± 10.2	24.7± 16.9	19.6 2.56	23.9± 1.34	27.4± 1.29	7.40± 2.15	5.93± 2.31	28.1± 4.50
Rhodospirillales	1.19± 1.15	0.88± 0.70	2.39± 1.21	1.81± 1.24	1.96± 0.21	1.43± 0.69	1.26± 0.80	0.01± 0.06	0.29± 0.14	0.09± 0.21	0.02± 1.56
Saprospirales	0.76± 0.23	0.36± 0.24	2.27± 1.15	1.04± 0.43	1.85± 1.01	1.56± 1.03	2.00± 0.23	1.69± 0.60	0.81± 0.40	0.45± 0.21	0.45± 0.12
Sphingobacteriales	2.30± 0.72	3.08± 085	5.40± 3.76	3.42± 2.40	2.42± 0.73	2.56± 0.32	2.68± 1.07	4.22± 0.21	1.02± 0.54	7.80± 2.56	0.13± 0.07
Sphingomonadales	0.01± 0.05	0.12± 0.21	0.37± 0.13	0.32± 0.21	0.31± 0.21	0.29± 0.7	0.61± 0.15	0.45± 0.21	0.12± 0.09	2.11± 0.30	0.21± 0.19
Stramenopiles	0.08± 0.10	0.43± 0.21	0.53± 0.21	0.60± 0.21	2.16± 1.87	0.65± 0.21	16.1± 6.54	5.41± 1.38	65.9± 12.3	2.31± 0.65	9.41± 2.32
Vibrionales	0.11± 0.06	1.91± 1.65	2.31± 1.05	1.74± 1.28	2.06± 0.01	3.12± 0.07	0.30± 0.21	9.09± 2.35	0.41± 0.23	0.02± 0.03	0.91± 0.32
Other	3.15± 0.87	2.87± 0.76	4.05± 0.76	3.56± 0.42	4.02± 2.09	3.78± 1.56	3.98± 1.89	1.65± 0.45	3.76± 0.32	3.54± 1.31	2.32± 0.56

Values correspond to the average and standard error for each of the taxa. Classes comprising <1% of the total number of sequences within a sample were simply classified as "Other".

4.6 Taxonomic abundance at the class level per day during winter

**Taxa**	**1**	**2**	**4**	**7**	**10**	**14**	**21**	**25**	**30**	**Ctr (30)**	**SW**
Actinomycetales	16.8± 3.45	16.8± 10.8	5.17± 1.21	24.1± 8.75	25.1± 7.65	13.5± 3.23	8.39± 2.91	17.9± 6.53	8.12± 2.89	5.47± 2.19	18.7± 3.21
Alteromonadales	3.69± 2.13	3.19± 1.57	2.19± 1.21	2.49± 1.24	2.87± 1.40	4.46± 2.13	3.20± 1.24	5.70± 2.31	5.43± 1.12	10.2± 1.91	7.98± 2.13
Bacteroidales	0.37± 0.25	0.23± 0.14	0.50± 0.21	0.28± 0.12	6.32± 1.56	0.34± 0.21	0.47± 0.16	0.54± 0.21	0.38± 0.16	0.23± 0.11	8.60± 2.45
Burkholderiales	1.04± 0.56	2.45± 1.45	1.43± 0.34	1.80± 0.54	1.12± 0.56	0.65± 0.39	1.51± 0.45	1.85± 0.43	0.62± 0.21	0.24± 0.12	1.36± 0.45
Campylobacterales	2.75± 0.23	0.90± 0.67	4.24± 1.25	1.52± 0.37	1.07± 0.95	2.76± 1.12	1.29± 0.45	1.28± 0.41	3.83± 1.21	0.52± 0.31	0.12± 0.05
Chroococcales	0.46± 0.21	0.54± 0.20	0.56± 0.18	0.67± 0.23	0.76± 0.23	0.92± 0.71	0.50± 0.19	1.31± 0.61	0.27± 0.19	0.27± 0.12	1.41± 0.43
Clostridiales	0.45± 0.21	2.32± 1.43	0.70± 0.31	0.88± 0.12	0.88± 0.45	0.59± 0.40	1.92± 0.54	0.81± 0.21	0.90± 0.32	0.90± 0.21	5.88± 1.77
Cytophagales	1.10±	1.31±	1.28±	8.95±	7.65±	6.74±	0.88±	1.40±	0.57±	0.57±	0.50±

	0.92	0.67	0.56	1.31	2.31	0.91	0.31	0.41	0.19	0.13	0.19
Deinococcales	0.01± 0.04	1.41± 0.12	1.53± 0.05	1.24± 0.76	1.35± 0.45	0.95± 0.23	0.81± 0.65	0.22± 0.12	0.32± 0.05	0.21± 0.06	0.92± 0.65
Flavobacteriales	8.83± 2.71	6.85± 1.58	11.4± 3.46	6.41± 4.95	5.86± 2.12	7.17± 2.24	7.13± 3.11	9.50± 2.31	6.82± 2.34	14.6± 3.21	6.38± 1.67
Methylococcales	0.20± 0.06	0.01± 0.04	0.05± 0.02	0.04± 0.21	0.08± 0.05	0.04± 0.04	1.01± 0.16	1.10± 0.80	0.53± 0.21	1.19± 0.21	2.63± 0.15
Mycoplasmatales	0.02± 0.01	0.01± 0.01	0.01± 0.06	0.02± 0.05	0.01± 0.04	0.00± 0.11	0.01± 0.01	0.01± 0.06	0.02± 0.05	0.01± 0.04	0.00± 0.11
Oceanospirillales	5.19± 2.11	3.55± 2.85	3.31± 1.42	2.72± 1.77	2.57± 0.32	2.59± 0.56	3.57± 1.09	4.58± 1.02	2.82± 0.31	23.8± 2.14	0.16± 0.07
Oscillatoriales	1.02± 0.77	2.13± 1.56	1.42± 0.35	8.55± 2.31	2.63± 0.43	1.87± 0.43	0.88± 0.31	3.05± 1.21	1.48± 0.32	0.44± 0.12	3.72± 2.13
Pseudomonadales	1.94± 1.23	1.13± 0.19	2.17± 0.21	1.12± 0.87	0.99± 0.31	0.96± 0.41	1.78± 0.51	2.26± 0.34	0.58± 0.19	1.45± 0.43	0.90± 0.45
Rhizobiales	3.56± 1.43	2.78± 0.76	2.28± 1.05	2.53± 1.04	2.79± 1.21	2.53± 0.67	1.34± 0.45	1.31± 0.45	4.31± 1.19	1.31± 0.19	2.27± 1.32
Rhodobacterales	31.9± 11.8	15.3 ± 6.37	34.3± 10.9	8.52± 2.02	17.0± 2.41	26.1± 3.21	27.2± 2.45	16.9± 1.76	28.3± 2.31	18.3± 2.54	11.7± 3.31
Rhodospirillales	1.54± 0.45	1.72± 0.71	0.81± 0.27	2.09± 1.02	1.44± 0.65	1.06± 0.41	0.15± 0.31	1.76± 0.45	0.81± 0.21	1.31± 0.21	1.10± 0.19
Saprospirales	1.65± 0.32	1.21± 0.19	1.74± 0.23	2.19± 0.54	0.97± 0.32	1.72± 0.32	1.15± 0.19	2.53± 0.43	1.12± 0.43	0.98± 0.19	1.41± 0.45
Sphingobacteriales	0.34± 0.21	0.45± 0.21	0.47± 0.19	0.89± 0.13	0.45± 0.41	0.32± 0.13	1.23± 0.56	2.40± 0.45	3.98± 1.09	0.15± 0.08	1.55± 0.45
Sphingomonodales	0.16± 0.05	2.08± 1.32	3.51± 1.21	3.45± 1.09	3.47± 1.31	3.90± 0.31	2.40± 0.67	3.12± 0.56	1.23± 0.56	2.08± 0.34	3.02± 1.21
Stramenopiles	2.80± 1.75	2.48± 0.56	1.38± 0.34	5.10± 2.76	1.46± 0.45	2.73± 0.31	3.75± 0.56	1.82± 0.45	3.18± 2.10	6.52± 3.31	3.99± 1.77
Vibrionales	0.91± 0.18	1.23± 0.78	3.32± 2.11	0.32± 0.10	0.10± 0.05	6.34± 0.66	3.92± 0.45	5.61± 1.09	3.25± 0.31	1.06± 0.45	2.00± 0.51
Other	3.41± 1.05	2.56± 5.34	1.89± 0.54	2.89± 1.98	3.41± 2.78	6.53± 2.09	2.07± 1.89	4.51± 3.11	2.11± 1.78	2.09± 1.34	1.32± 0.56

Values correspond to the average and standard error for each of the taxa. Classes comprising <1% of the total number of sequences within a sample were simply classified as “Other”.
