# Supplementary material for: Distinct Temporal Succession of Bacterial Communities in Early Marine Biofilms in a Portuguese Atlantic Port
Source: Front Microbiol. 2020 Aug 11;11:1938. doi: 10.3389/fmicb.2020.01938 (PMC7432428; doi:10.3389/fmicb.2020.01938)
Supplement: TABLE S5 — Taxonomic abundance at the family level for the different treatments. [file Table_5.pdf]

Supplementary Table 5: Taxonomic abundance at the family level for the different treatments

5.1 Taxonomic abundance at the class level for the total samples

Taxa	Total
Alteromonodaceae	5.36 ± 3.90
Clostridiaceae	0.17 ± 0.11
Cytophagaceae	1.21 ± 1.04
Deinococcaceae	1.45 ± 0.73
Flavobacteriaceae	10.9 ± 4.31
Halomonodaceae	7.43 ± 2.68
Helicobacteraceae	1.04 ± 0.40
Hyphomonadaceae	1.77 ± 0.98
Moraxellaceae	0.75 ± 0.32
Nocardioidaceae	4.93 ± 2.07
Oceanospirillaceae	0.81 ± 0.36
Phormidiaceae	1.80 ± 1.57
Propionibacteriaceae	0.53 ± 0.58
Pseudoalteromonadaceae	1.37 ± 0.46
Rhodobacteraceae	22.1 ± 2.25
Saprospiraceae	1.30 ± 0.17
Sphingomonadaceae	1.70 ± 0.53
Vibrionaceae	1.56 ± 0.44
Xenococcaceae	1.18 ± 0.23
Other	23.67 ± 2.61

Values correspond to the average and standard error for each of the taxa. Families comprising <1% of the total number of sequences within a sample were simply classified as "Other".

5.2 Taxonomic abundance per season at the family level

Taxa	Spring	Winter
Alteromonodaceae	4.32 ± 1.23	2.05 ± 1.09
Clostridiaceae	1.87± 0.32	0.32 ± 0.05
Cytophagaceae	0.50± 0.21	2.25 ± 1.13
Deinococcaceae	1.55 ± 0.56	1.78 ± 0.43
Flavobacteriaceae	10.8 ± 4.31	9.49 ± 5.43
Halomonodaceae	2.10 ± 0.45	2.98 ± 1.29
Helicobacteraceae	0.76 ± 0.32	0.95 ± 0.41
Hyphomonadaceae	1.51 ± 0.31	1.32 ± 0.26
Moraxellaceae	0.82 ± 0.34	0.71 ± 0.34
Mycoplasmaceae	0.53 ± 0.22	0.43 ± 0.10
Nocardioidaceae	1.03 ± 0.33	5.71 ± 2.13

Oceanospirillaceae	1.13 ± 0.56	1.00 ± 1.05
Phormidiaceae	1.73 ± 0.43	0.94 ± 0.45
Propionibacteriaceae	0.23 ± 0.12	1.16 ± 0.46
Pseudoalteromonadaceae	1.11 ± 0.54	2.34 ± 1.21
Rhodobacteraceae	20.9 ± 14.3	23.6 ± 11.4
Saprospiraceae	1.43 ± 0.34	0.81 ± 0.23
Sphingomonadaceae	1.31 ± 0.96	2.08 ± 1.23
Vibrionaceae	1.10± 0.43	1.87 ± 0.56
Xenococcaceae	0.98 ± 0.41	1.28 ± 0.21
Other	22.46 ± 0.41	24.12 ± 0.19

Values correspond to the average and standard error for each of the taxa. Families comprising <1% of the total number of sequences within a sample were simply classified as "Other".

5.3 Taxonomic abundance per treatment at the family level

**Taxa**	**Seawater**	**Plates without anti-corrosion paint**	**Plates with anti-corrosion paint**
Alteromonodaceae	9.33 ± 2.31	1.50 ± 0.45	5.36 ± 0.86
Clostridiaceae	2.23 ± 1.45	5.05 ± 0.144	0.55 ± 0.16
Cytophagaceae	0.77 ± 0.15	2.47 ± 1.23	0.57 ± 0.19
Deinococcaceae	1.88 ± 0.18	1.97 ± 1.47	0.61 ± 0.31
Flavobacteriaceae	2.77 ± 0.18	10.2 ± 8.47	15.7 ± 7.19
Halomonodaceae	10.7 ± 5.14	0.35 ± 0.19	11.4 ± 0.72
Helicobacteraceae	3.41 ± 0.78	2.45 ± 1.98	0.21 ± 0.05
Hyphomonadaceae	1.21 ± 0.97	1.27 ± 0.91	2.84 ± 1.01
Moraxellaceae	3.03 ± 1.03	2.68 ± 1.21	1.67 ± 0.87
Nocardioidaceae	2.25 ± 1.13	5.30 ± 2.35	2.74 ± 0.77
Oceanospirillaceae	0.50 ± 0.23	1.23 ± 0.57	0.71 ± 0,43
Phormidiaceae	1.16 ± 0.56	1.53 ± 0.79	0.47 ± 0.21
Propionibacteriaceae	0.56 ± 0.27	1.22 ± 0.37	0.35 ± 0.11
Pseudoalteromonadaceae	0.87 ± 0.54	1.20 ± 0.70	1.90 ± 0.99
Rhodobacteraceae	24.8 ± 8.95	20.9 ± 13.3	20.7 ± 0.51
Saprospiraceae	1.15 ± 0.89	1.14 ± 0.31	1.44 ± 0.35
Sphingomonadaceae	1.52 ± 0.96	2.25 ± 1.14	1.31 ± 0.26
Vibrionaceae	0.41 ± 0.15	1.26 ± 0.65	1.92 ± 0.26
Xenococcaceae	2.02 ± 0.10	0.91 ± 0.56	0.32 ± 0.26
Other	23.01 ± 2.09	22.18 ± 5.31	21.89 ± 0.56

Values correspond to the average and standard error for each of the taxa. Families comprising <1% of the total number of sequences within a sample were simply classified as "Other".

5.4 Taxonomic abundance per day for both seasons at the family level

**Taxa**	**1**	**2**	**4**	**7**	**10**	**14**	**21**	**25**	**30**
Alteromonodaceae	1.34± 0.16	0.90± 0.21	0.82± 0.41	0.70± 0.28	1.16± 0.32	1.05± 0.65	3.67± 1.28	1.30± 0.32	7.75± 3.19

Clostridiaceae	0.54± 0.16	0.31± 0.10	1.22± 0.90	1.31± 0.28	1.04± 0.21	0.98± 0.21	1.78± 0.45	1.34± 0.34	1.87± 0.56
Cryomorphaceae	0.14± 0.16	1.22± 0.31	1.32± 1.04	1.39± 0.41	1.14± 0.50	0.96 ± 0.64	1.26 ± 0.82	1.10 ± 0.34	2.06 1.19
Cytophagaceae	0.72± 0.26	3.82± 1.08	1.12± 0.08	2.49± 0.68	6.34± 3.11	7.46± 0.64	0.38± 0.12	0.60± 0.12	0.36± 0.19
Deinococcaceae	0.75± 0.15	2.82± 1.08	0.92± 0.04	1.60± 0.38	5.35± 0.21	5.16± 0.65	0.50± 0.28	0.80± 0.12	0.46± 0.19
Flavobacteriaceae	0.35± 0.14	4.33± 1.28	7.43± 1.28	8.19± 4.28	6.95± 0.31	6.16± 0.89	20.5± 13.3	13.6± 8.12	17.7± 7.19
Halomonodaceae	0.62± 0.16	0.23± 0.15	0.33± 0.18	0.19± 0.08	0.28± 0.21	0.16± 0.05	0.23± 0.19	0.70± 0.12	10.8± 6.91
Helicobacteraceae	1.82± 0.69	1.43± 0.76	4.43± 0.76	2.75± 1.23	1.74± 1.33	2.25± 0.84	2.75± 0.13	1.20± 0.12	0.85± 0.31
Hyphomonadaceae	1.12± 0.59	0.53± 0.21	1.23± 0.65	1.82± 0.88	1.02± 0.21	1.16± 0.65	1.44± 1.04	2.43± 1.32	1.81± 1.45
Moraxellaceae	1.82± 0.60	1.43± 0.23	1.42± 0.71	1.09± 0.65	0.87± 0.21	1.56± 0.58	2.02± 0.41	0.78± 0.32	1.09± 0.34
Nocardioidaceae	10.8± 3.41	16.3± 7.43	3.48± 2.22	9.64± 7.54	5.59± 2.31	8.75± 3.22	2.04± 1.07	3.80± 1.48	1.66 ± 2.06
Oceanospirillaceae	1.42± 0.69	0.98± 0.32	1.72± 0.73	0.62± 0.23	0.91± 0.67	1.12± 0.61	1.38± 1.42	1.90± 1.25	0.54± 0.21
Phormidiaceae	1.51± 0.23	2.14± 1.21	1.52± 1.15	6.23± 4.28	2.67± 0.44	1.92± 0.45	1.85± 0.45	3.06± 1.74	1.66± 1.06
Propionibacteriaceae	0.51± 0.38	0.72± 0.14	0.32± 0.15	0.62± 0.17	0.77± 0.34	2.60± 0.40	0.54± 0.32	0.89± 0.32	0.44± 0.32
Pseudoalteromonadaceae	0.32± 0.19	0.22± 0.05	3.16± 1.41	0.32± 0.11	0.28± 0.10	0.21± 0.04	1.24± 0.32	3.56± 0.80	1.54± 1.06
Rhodobacteraceae	0.72± 0.68	15.3± 7.86	27.4± 14.7	19.6± 9.41	14.1± 7.98	19.5± 13.8	22.4± 9.57	28.2± 11.3	19.4± 2.06
Saprospiraceae	1.61± 0.45	0.74± 0.71	1.33± 0.17	3.17± 1.23	1.41± 0.80	1.41± 0.80	1.13± 0.37	1.80± 0.30	0.94 ± 0.20
Sphingomonadaceae	1.42± 0.31	6.47± 2.46	2.91± 2.02	3.57± 0.45	1.96± 1.33	3.33± 1.02	0.71± 0.49	1.41± 0.50	1.24± 0.21
Vibrionaceae	0.32± 0.25	0.37± 0.16	0.91± 0.48	0.37± 0.14	0.10± 0.05	0.23± 0.02	3.61± 0.45	4.20± 0.30	1.64± 1.06
Xenococcaceae	0.31± 0.19	0.45± 0.15	1.23± 0.67	6.23± 2.02	0.23± 0.11	1.24± 0.31	2.41± 1.06	3.41± 1.41	0.27± 0.09
Other	22.3± 10.2	16.7± 3.45	19.2± 3.45	21.9± 2.98	15.6± 3.45	13.7± 3.42	18.9± 10.9	21.0± 9.76	14.5± 7.86

Values correspond to the average and standard error for each of the taxa. Families comprising <1% of the total number of sequences within a sample were simply classified as "Other".

5.5 Taxonomic abundance at the family level per day during spring

**Taxa**	**1**	**2**	**4**	**7**	**10**	**14**	**21**	**25**	**30**	**Ctr (30)**	**SW**
Anaplasmataceae	0	0	0	0	0	0	0.004± 0.002	0	0	0	0.01± 0.02
Clostridiaceae	12.1± 2.34	10.9± 4.31	13.1± 3.42	4.52± 2.31	12.1± 2.34	4.58± 0.90	0.001± 0.002	0.08± 0.10	0.12± 0.06	4.59± 1.14	0.06± 0.04

Cryomorphaceae	7.11± 2.56	21.5± 4.67	5.55± 0.89	13.6± 1.98	14.9± 3.67	15.2± 2.21	4.21± 1.23	6.50± 2.12	3.30± 1.45	3.21± 1.23	3.30± 1.45
Cytophagaceae	0.3 ± 0.12	0.90± 0.21	0.40± 0.20	0.50± 0.13	0.45± 0.24	2.11± 0.98	0.21 ± 0.09	0.32 ±0.12	0.20± 0.05	0.19± 0.05	0.10± 0.02
Deinococcaceae	1.21± 0.43	0.60± 0.32	0.50± 0.23	2.21 ± 0.21	1.71± 0.41	2.02± 1.09	1.41 ± 0.43	1.31± 0.34	0.98± 0.25	0.80± 0.43	0.04± 0.03
Flavobacteriaceae	0.70± 0.21	2.81± 0.34	3.43± 0.67	7.69 ± 2.34	4.23± 2.31	8.20± 2.31	9.24± 3.13	10.6 ± 1.89	2.03± 1.45	22.6± 11.1	0.02± 0.01
Halomonodaceae	0.90± 0.34	1.31± 0.34	1.03± 0.23	0.80± 0.12	0.50± 0.09	0.61± 0.13	0.60± 0.04	1.12± 0.06	0.30± 0.19	0.22± 0.10	0.32± 0.09
Helicobacteraceae	0.25± 0.12	0.31± 0.18	0.48± 0.19	3.25± 1.21	1.75± 0.45	0.54 ± 0.21	1.14± 0.23	0.44± 0.15	0.25± 0.12	0.05± 0.03	0
Hyphomonadaceae	0.32± 0.11	0.25± 0.11	0.15± 0.09	1.13± 0.21	1.01± 0.12	1.16± 0.21	2.15± 1.23	2.65± 0.56	0.36± 0.14	0.09± 0.03	0.003± 0.002
Moraxellaceae	1.42± 0.45	1.41± 0.31	5.02± 0.89	5.12± 1.12	1.17± 0.90	0.14± 0.05	0.17± 0.03	0.001± 0.001	0.004± 0.002	0.14± 0.09	4.61± 2.35
Nocardioidaceae	1.09± 0.43	3.08± 1.45	4.28± 3.19	4.65 ± 0.77	0.005± 0.002	0.003± 0.002	0.001± 0.001	1.42 ± 0.34	0.001± 0.002	0.02± 0.02	0
Oceanospirillaceae	17.3± 5.67	18.2± 5.44	7.95± 2.31	1.43 ± 0.74	1.19± 0.12	0.24± 0.11	0.22± 0.04	0.54 ±0.12	0.06 ± 0.05	0.002± 0.002	0.0001 ± 0.0001
Phormidiaceae	0.21± 0.11	0.45± 0.41	2.28± 0.45	0.55± 0.12	0.44 ± 0.11	1.88± 0.56	2.02± 0.73	1.19± 0.34	0.73± 0.45	0.05± 0.03	0
Propionibacteriaceae	4.00± 1.25	4.01± 0.65	1.05± 0.45	3.74 ± 1.23	3.66 ± 2.01	0.14± 0.06	0.01± 0.03	0.03 ±0.02	0.05± 0.03	0.88± 0.41	0
Pseudoalteromonadaceae	0.10± 0.05	2.12± 1.24	17.0± 2.34	1.42± 1.14	0.47± 0.18	0.0001 ± 0.0001	0.15± 0.09	8.65 ± 2.34	0.18± 0.12	0.013 ± 0.05	0
Rhodobacteraceae	0.32±0 .11	1.18 ±0.62	4.91 ±0.87	17.1± 10.2	21.1 ± 5.67	43.5± 16.7	21.8± 14.3	24.1± 9.91	7.04± 2.45	7.26± 3.41	0
Saprospiraceae	1.61± 0.45	0.74± 0.71	1.33± 0.17	3.17± 1.23	1.41± 0.80	1.41± 0.60	1.13± 0.37	1.80 ± 0.30	0.94 ± 0.20	1.09± 0.45	0.98± 0.65
Sphingomonadaceae	1.77± 0.45	2.18± 0.31	0.90± 0.23	2.95 ± 1.45	1.95± 0.21	0.94± 0.34	1.08± 0.22	1.44± 0.34	0.22± 0.14	0	5.62± 2.31
Vibrionaceae	0	0	10.0± 3.41	0.31± 0.12	0.02± 0.02	0.01± 0.03	0.16± 0.09	0.44 ±0.31	0.23± 0.02	0.01± 0.05	0.10± 0.05
Xenococcaceae	0.45± 0.12	0.41± 0.07	6.10± 2.31	0.45± 0.23	0.70± 0.12	1.65± 0.32	1.44± 0.56	0.62± 0.28	0.63± 0.13	0.28± 0.12	0.27± 0.15
Other	25.1± 9.41	15.2± 5.45	10.2± 5.31	11.9± 4.98	11.6± 5.67	10.7± 4.56	14.2± 6.76	15.1± 4.12	10.5± 5.63	12.4± 6.41	12.3± 4.31

Values correspond to the average and standard error for each of the taxa. Ctr (30) refers to plates with anti-corrosion paint; SW refers to seawater; Families comprising <1% of the total number of sequences within a sample were simply classified as "Other".

5.6 Taxonomic abundance at the family level per day during winter

**Taxa**	**1**	**2**	**4**	**7**	**10**	**14**	**21**	**25**	**30**	**Ctr (30)**	**SW**
Anaplasmataceae	0.01± 0.03	0	0	0	0.001± 0.001	0	0	0	0	0	35.9± 7.89

Clostridiaceae	0.29± 0.15	1.22± 0.45	0.32± 0.19	0.52± 0.23	0.12± 0.04	0.45± 0.21	1.06± 0.31	3.93± 1.74	1.04± 0.45	0.54± 0.19	4.48± 2.10
Cryomorphaceae	7.11± 2.34	6.11± 2.31	5.51± 2.23	13.6± 3.56	14.9± 4.56	15.2± 9.87	4.34± 0.56	6.51± 2.31	3.31± 1.32	3.31± 1.23	3.30± 1.12
Cytophagaceae	0.31± 0.19	0.90± 0.43	0.40± 0.32	0.51± 0.16	0.53± 0.13	2.12± 1.24	0.21± 0.15	0.31± 0.19	0.11± 0.09	0.10± 0.08	0.12± 0.09
Deinococcaceae	1.21± 0.45	0.60± 0.45	0.50± 0.21	2.21± 0.87	1.71± 0.08	1.91± 0.34	1.41± 0.31	1.30± 0.49	0.08± 0.05	0.80 ± 0.65	0.80± 0.12
Flavobacteriaceae	0.89± 0.34	5.42± 2.31	9.57± 4.21	4.81± 0.23	3.44± 2.11	5.60± 2.46	17.3± 9.11	14.8± 6.71	17.7± 4.56	12.9± 4.31	5.52± 1.43
Halomonodaceae	0.90± 0.21	1.31± 0.56	1.01± 0.21	0.80± 0.21	0.52± 0.12	0.60± 0.17	0.60± 0.05	1.11± 0.31	0.31± 0.15	0.30± 0.09	0.21± 0.08
Helicobacteraceae	2.62± 1.05	1.20 ± 0.27	3.97± 1.45	0.57± 0.21	1.00± 0.23	2.64± 0.31	1.20± 0.09	0.01± 0.12	3.49± 1.21	0.27± 0.06	0.10± 0.05
Hyphomonadaceae	1.12± 0.56	1.29± 0.31	1.35± 0.45	0.86± 0.21	0.34± 0.18	1.00± 0.21	1.12± 0.43	0.93± 0.25	0.74± 0.26	1.51± 0.09	0.79± 0.21
Moraxellaceae	0.76± 0.32	0.68± 0.31	1.39± 0.45	0.56± 0.15	0.42± 0.05	0.56± 0.23	1.07± 0.41	1.40± 0.67	0.42± 0.19	0.68± 0.23	012± 0.09
Nocardioidaceae	6.42± 1.23	9.85± 4.71	3.83± 2.56	14.5± 2.31	6.94± 5.92	2.75± 1.19	4,78± 2.22	7.30± 2.12	1.47± 1.34	0.01± 0.02	6.75± 2.21
Oceanospirillaceae	1.70± 0.43	0.34± 0.12	1.95± 0.41	1.21± 0.32	0.98± 0.70	0.42± 0.62	1.38± 1.12	0.89± 0.56	0.92± 0.34	5.04± 2.31	0.27± 0.13
Phormidiaceae	1.02± 0.32	2.13± 1.35	0.52± 0.19	6.22± 3.21	2.62± 1.06	1.87± 1.61	0.37± 0.24	3.05± 1.24	0.99± 0.21	0.44± 0.19	3.47± 1.06
Propionibacteriaceae	6.23± 3.45	2.14± 0.13	0.26± 0.12	2.19± 1.09	4.09± 2.11	6.51± 4.09	2.69± 1.45	4.56± 2.31	4.49± 2.27	2.68± 1.31	14.8± 2.31
Pseudoalteromonadaceae	0.51± 0.15	0.34± 1.08	2.73± 1.21	0.15± 0.09	0.21± 0.15	0.35± 0.17	1.03± 0.91	1.04± 0.21	0.64± 0.21	2.37± 1.15	1.80± 0.45
Rhodobacteraceae	24.6 ± 11.7	14.1 ± 5.91	27.0± 4.56	13.0± 2.34	16.6± 2.32	24.9± 12.1	16.4± 7.71	14.8± 7.67	22.8± 10.2	16.7± 8.80	10.9± 3.31
Saprospiraceae	1.71 ± 0.45	0.84 ± 0.51	1.12± 0.21	2.56 ± 1.07	1.21 ± 0.50	1.41 ± 0.60	1.01 ± 0.41	1.50± 0.20	0.81± 0.12	1.12± 0.12	1.26± 0.74
Sphingomonadaceae	1.61± 0.69	3.15± 0.05	3.87± 2.14	7.43± 2.31	0.65± 0.32	3.45± 1.02	0.75± 0.61	0.15± 0.12	2.06± 1.21	0.93± 0.85	1.34± 0.26
Vibrionaceae	0.40± 0.21	0.59± 0.19	0.41± 0.19	0.18± 0.10	0.12± 0.09	0.22± 0.07	3.26± 1.45	2.07± 1.21	3.09± 1.05	0.88± 0.44	0.25± 0.10
Xenococcaceae	0.36± 0.19	2.25± 0.41	0.89± 0.24	1.56± 0.87	0.43± 0.21	0.92± 0.15	0.15± 0.10	2.55± 0.56	0.30± 0.12	0.27± 0.12	1.35± 0.23
Other	13.1± 3.23	10.2± 2.14	13.6± 6.70	10.2± 5.61	19.6± 6.23	11.2± 4.56	10.2± 4.51	10.2± 7.89	12.1± 4.51	10.4± 2.13	10.1± 5.43

Values correspond to the average and standard error for each of the taxa. Ctr (30) refers to plates with anti-corrosion paint; SW refers to seawater; Families comprising <1% of the total number of sequences within a sample were simply classified as "Other".
