# Supplementary material for: Distinct Temporal Succession of Bacterial Communities in Early Marine Biofilms in a Portuguese Atlantic Port
Source: Front Microbiol. 2020 Aug 11;11:1938. doi: 10.3389/fmicb.2020.01938 (PMC7432428; doi:10.3389/fmicb.2020.01938)
Supplement: TABLE S6 — Taxonomic abundance at the genus level for the different treatments. [file Table_6.pdf]

Supplementary Table 6: Taxonomic abundance at the genus level for the different treatments

6.1 Taxonomic abundance at the genus level for the total samples

Taxa	Total
Candidatus Portiera	0.47 ± 0.29
Chroococcidiopsis	0.78 ± 0.73
Clostridium	2.81 ± 2.39
Friedmanniella	4.04 ± 2.15
Glaciecola	0.96 ± 0.54
Hymenobacter	1.30 ± 0.87
Loktanella	1.72 ± 0.70
Mycoplasma	0.51 ± 5.02
Octadecabacter	4.17 ± 1.13
Oleispira	1.97 ± 2.37
Phormidium	1.41 ± 0.65
Propionibacterium	2.77 ± 0.89
Pseudoalteromonas	1.94 ± 2.29
Proteus	0.31 ± 0.38
Sediminicola	0.32 ± 0.18
Sphingomonas	1.69 ± 0.56
Sulfurimonas	0.99 ± 0.58
Other	37.3 ± 12.3

Values correspond to the average and standard error for each of the taxa. Orders comprising <1% of the total number of sequences within a sample were simply classified as "Other".

6.2 Taxonomic abundance per season at the genus level

Taxa	Spring	Winter
Candidatus Portiera	0.15 ± 0.60	0.79 ± 0.12
Chroococcidiopsis	0.56 ± 0.21	1.21 ± 0.19
Clostridium	1.25 ± 0.26	4.41 ± 0.79
Friedmanniella	6.17 ± 2.01	1.90 ± 1.23
Glaciecola	0.58 ± 0.23	1.33 ± 0.19
Hymenobacter	2.26 ± 1.21	0.34 ± 0.32
Loktanella	2.44 ± 1.03	1.00 ± 0.56
Mycoplasma	0.006 ± 0.003	1.00 ± 0.12
Octadecabacter	4.03 ± 0.98	4.30 ± 0.34
Oleispira	1.05 ± 0.31	2.89 ± 1.43
Phormidium	2.00 ± 0.45	0.82 ± 0.23
Propionibacterium	3.73 ± 1.31	3.70 ± 0.89

Pseudoalteromonas	0.65 ± 0.21	3.28 ± 1.09
Proteus	0.07 ± 0.02	0.56 ± 0.23
Sediminicola	0.16 ± 0.29	0.47 ± 0.18
Sphingomonas	1.85 ± 0.56	1.54 ± 0.45
Sulfurimonas	1.40 ± 0.28	0.57 ± 0.29
Other	37.3 ± 12.3	28.1 ± 9.12

Values correspond to the average and standard error for each of the taxa. Orders comprising <1% of the total number of sequences within a sample were simply classified as "Other".

6.3 Taxonomic abundance per treatment at the class level

**Taxa**	**Seawater**	**Plates without anti-corrosion paint**	**Plates with anti-corrosion paint**
Candidatus Portiera	3.07 ± 1.21	0.27 ± 0.09	0.14 ± 0.03
Chroococcidiopsis	1.21 ± 0.23	1.09 ± 0.81	0.17 ± 0.02
Clostridium	4.47 ± 2.12	4.41 ± 2.68	2.49 ± 0.46
Friedmanniella	5.60 ± 2.35	1.78 ± 1.63	0.98 ± 0.88
Glaciecola	6.84 ± 3.21	0.12 ± 0.11	0.32 ± 0.31
Hymenobacter	0.26 ± 0.11	0.38 ± 0.58	0.28 ± 0.22
Loktanella	0.50 ± 0.15	1.16 ± 0.83	1.95 ± 1.45
Mycoplasma	0	7.26 ± 6.78	0.07 ± 0.05
Octadecabacter	7.27 ± 4.44	3.88 ± 3.57	1.68 ± 1.28
Oleispira	0.07 ± 0.08	3.54 ± 3.14	3.12 ± 2.20
Phormidium	2.30 ± 0.24	0.74 ± 0.52	0.29 ± 0.22
Propionibacterium	7.40 ± 2.56	1.73 ± 1.59	2.39 ± 2.14
Pseudoalteromonas	1.68 ± 0.21	3.82 ± 2.56	0.63 ± 0.46
Proteus	0.05 ± 0.02	0.71 ± 0.64	0.27 ± 0.21
Sediminicola	2.28 ± 2.22	0.07 ± 0.05	0.07 ± 0.05
Sphingomonas	0.78 ± 0.14	1.77 ± 0.77	0.45 ± 0.30
Sulfurimonas	0.03 ± 0.01	0.69 ± 0.65	0.09 ± 0.06
Other	45.6 ± 6.71	42.1± 2.34	41.0± 7.89

Values correspond to the average and standard error for each of the taxa. Classes comprising <1% of the total number of sequences within a sample were simply classified as "Other".

6.4 Taxonomic abundance per day for both seasons at the order level

**Taxa**	**1**	**2**	**4**	**7**	**10**	**14**	**21**	**25**	**30**
Candidatus Portiera	0.48± 0.07	0.35± 0.18	0.13± 0.11	0.13± 0.08	0.51± 0.47	0.17± 0.08	0.12± 0.09	0.09± 0.06	0.04± 0.03
Chroococcidiopsis	0.07± 0.03	0.80± 0.31	3.21± 2.68	2.38± 1.53	0.20± 0.01	0.14± 0.10	0.22± 0.20	0.68± 0.49	0.12± 0.03

Clostridium	7.19± 5.32	8.75 ± 7.29	1.25± 1.03	1.21± 1.01	8.22± 5.82	0.12± 0.09	0.70± 0.52	2,73± 1.96	0.64± 0.52
Friedmanniella	5.81± 3.99	4.50 ± 3.82	3.85 ± 0.38	11.1± 9.32	6.76± 5.16	6.65± 5.25	1.30± 0.56	4.20± 3.96	1.05± 0.57
Glaciecola	0.31± 0.22	0.60 ± 0.47	0.37± 0.14	0.77± 0.64	0.32± 0.31	0.32± 0.31	0.16± 0.16	0.09± 0.07	0.28± 0.28
Hymenobacter	0.58± 0.41	0.97± 0.81	1.35± 0.65	4.08± 2.89	3.94± 3.13	4.18± 2.96	0.12± 0.02	0.45± 0.38	0.23± 0.18
Loktanella	2.58± 1.90	1.25± 0.95	1.28± 0.82	1.48± 0.45	1.83± 0.40	2.33± 0.30	2.10± 0.96	2.40± 1.04	2.27± 2.22
Mycoplasma	19.9± 14.1	16.0± 11.3	0.41± 0.29	1.22± 0.86	8.41± 6.01	0.08± 0.02	0.008± 0.005	0.004± 0.002	0.05± 0.04
Octadecabacter	3.99± 3.10	6.55± 4.91	6.33± 4.98	4.38± 1.83	3.97± 1.07	7.01± 5.37	4.22± 1.43	3.93± 2.93	2.74± 1.76
Oleispira	12.0± 9.12	6.55± 4.91	2.16± 1.99	0.74± 0.20	0.67± 0.65	0.77± 0.58	0.74± 0.58	0.68± 0.66	0.57± 0.44
Phormidium	0.68± 0.48	2.07± 0.02	0.86± 0.72	4.10± 3.27	1.69± 1.40	1.96± 0.90	0.72± 0.60	3.21± 2.28	0.32± 0.12
Propionibacterium	2.33± 2.29	4.87± 3.95	0.62± 0.59	1.24± 0.93	1.43± 1.38	0.23± 0.20	2.81± 2.00	5.25± 3.23	3.15± 2.27
Pseudoalteromonas	0.28± 0.23	1.55± 1.31	10.5± 9.26	4.04± 2.97	0.33± 0.20	0.13± 0.10	0.43± 0.41	5.75± 4.42	0.27± 0.13
Proteus	0.92± 0.68	1.08± 0.97	0.38± 0.28	0.07± 0.01	1.41± 1.00	0.002± 0.002	0.06± 0.03	0.10± 0.08	0.04± 0.03
Sediminicola	0.23± 0.16	0.55± 0.39	0.08± 0.06	0.09± 0.03	0.18± 0.13	0.16± 0.11	0.01± 0.01	0.10± 0.008	0.04± 0.02
Sphingomonas	1.83± 0.35	2.31± 0.35	4.51± 3.25	3.36± 1.34	1.69± 1.58	1.63± 1.61	0.72± 0.52	0.84± 0.61	0.74± 0.16
Sulfurimonas	1.48± 1.05	0.34± 0.24	2.27± 1.92	2.60± 2.42	0.86± 0.11	0.48± 0.44	1.18± 1.10	0.55± 0.33	2.01± 1.47
Other	23.4± 12.1	32.3± 9.87	26.5± 12.3	32.1± 16.3	28.3± 12.7	30.1± 19.2	23.2± 9.87	26. 1± 7.90	20.3± 9.87

Values correspond to the average and standard error for each of the taxa. Classes comprising <1% of the total number of sequences within a sample were simply classified as "Other".

6.5 Taxonomic abundance at the class level per day during spring

**Taxa**	**1**	**2**	**4**	**7**	**10**	**14**	**21**	**25**	**30**	**Ctr (30)**	**SW**
Candidatus Portiera	0.52± 0.19	0.48± 0.18	0.02± 0.01	0.07± 0.03	0.83± 0.18	0.23± 0.08	0.18± 0.09	0.14± 0.08	0.02± 0.01	0.17± 0.09	6.06± 2.10
Chroococcidiopsis	0.05± 0.02	0.58± 0.21	4.95± 1.97	3.47± 1.26	0.20± 0.05	0.07± 0.05	0.35± 0.21	0.002± 0.002	0.14± 0.06	0.19± 0.06	1.13± 0.21
Clostridium	10.4± 4.31	13.5± 2.31	1.92± 0.43	1.92± 0.51	11.6± 2.09	0.18± 0.09	0.03± 0.04	0.03± 0.06	0.07± 0.05	4.44± 2.22	4.47± 1.19
Friedmanniella	2.98± 1.21	0.64± 0.21	4.13± 2.12	4.49± 2.12	0.38± 0.16	0.54± 0.23	0.91± 0.34	1.39± 0.37	0.64± 0.39	0.36± 0.15	4.48± 1.09

Glaciecola	0.30± 0.11	0.05± 0.02	0.28± 0.12	0.32± 0.10	0.08± 0.05	0.11± 0.06	0.05± 0.03	0.04± 0.05	0.08± 0.04	0.01± 0.03	13.6± 2.54
Hymenobacter	0.07± 0.02	0.39± 0.17	1.81± 0.34	0.01± 0.02	0.35± 0.12	0.005± 0.004	0.13± 0.06	0.72± 0.21	0.10± 0.05	0.02± 0.02	0.26± 0.13
Loktanella	0.07± 0.02	0.07± 0.02	0.70± 0.24	1.80± 0.25	1.54± 0.41	2.54± 1.31	1.42± 0.51	1.66± 0.43	0.70± 0.18	0.08± 0.04	0.50± 0.14
Mycoplasma	28.1± 3.11	22.7± 3.12	0.57± 0.29	1.73± 0.23	0.96± 0.54	0.10± 0.06	0.001± 0.002	0.01± 0.03	0.07± 0.04	0.11± 0.06	0
Octadecabacter	0.26± 0.12	0.24± 0.11	0.50± 0.12	0.60± 0.21	4.72± 1.87	10.8± 4.61	5.24± 2.11	6.00± 2.34	1.50± 0.45	0.08± 0.03	12.5± 4.31
Oleispira	17.7± 3.31	9.55± 2.31	3.52± 1.12	5.67± 2.12	0.18± 0.09	0.03± 0.02	0.06± 0.05	0.21± 0.06	0.03± 0.02	0.02± 0.04	0.01± 0.05
Phormidium	0.02± 0.01	2.06± 1.19	1.37± 0.23	0.36± 0.11	0.21± 0.13	1.32± 0.45	1.15± 0.56	0.008± 0.004	0.23± 0.10	0.78± 0.31	2.30± 1.14
Propionibacterium	3.95± 2.11	7.67± 2.19	1.04± 0.21	0.58± 0.13	2.39± 0.56	0.09± 0.05	0.01± 0.03	0.02± 0.05	0.04± 0.02	0.88± 0.12	0.10± 0.04
Pseudoalteromonas	0.11± 0.06	2.41± 0.56	16.7± 4.32	5.84± 1.17	0.47± 0.21	0.06± 0.03	0.15± 0.08	8.49± 2.67	0.18± 0.09	0.01± 0.05	1.68± 0.45
Proteus	1.33± 0.31	1.73± 0.61	0.55± 0.12	0.08± 0.04	2.00± 0.41	0.0005± 0.0004	0.001± 0.002	0.004± 0.002	0.0005 0.004	0.40± 0.19	0.06± 0.03
Sediminicola	0.02± 0.01	0.01± 0.02	0.01± 0.03	0.07± 0.03	0.28± 0.12	0.28± 0.14	0.0003± 0.0002	0.03± 0.02	0.05± 0.03	0.003± 0.005	4.57± 2.89
Sphingomonas	2.07± 1.21	2.07± 0.19	6.81± 0.34	2.41± 1.28	0.39± 0.17	0.39± 0.21	1.08± 0.21	0.01± 0.03	0.63± 0.25	0.23± 0.13	0.78± 0.20
Sulfurimonas	0.007± 0.004	0.004± 0.002	0.31± 0.12	4.31± 2.19	0.93± 0.15	0.93± 0.31	0.40± 0.19	0.14± 0.07	0.05± 0.03	0.05± 0.03	0.03± 0.02
Other	20.2± 10.1	23.3± 0.98	16.3± 10.4	12.1± 8.91	18.9± 10.1	25.1± 14.1	21.2± 7.87	22. 4± 4.80	18.5± 5.67	13.2± 4.67	10.2± 4.56

Values correspond to the average and standard error for each of the taxa. Classes comprising <1% of the total number of sequences within a sample were simply classified as "Other".

6.6 Taxonomic abundance at the class level per day during winter

**Taxa**	**1**	**2**	**4**	**7**	**10**	**14**	**21**	**25**	**30**	**Ctr (30)**	**SW**
Candidatus Portiera	0.43± 0.21	0.23± 0.12	0.20± 0.09	0.18± 0.07	0.11± 0.05	0.21± 0.06	0.008± 0.004	0.05± 0.04	0.05± 0.03	0.11± 0.05	0.07± 0.04
Chroococcidiopsis	0.09± 0.05	1.03± 0.45	0.41± 0.18	1.30± 0.45	0.21± 0.09	0.72± 0.17	0.04± 0.02	0.97± 0.45	0.10± 0.05	0.15± 0.09	1.13± 0.45
Clostridium	0.23± 0.13	1.10± 0.31	0.18± 0.09	0.49± 0.21	0.005 ±0.004	0.39± 0.14	1.02± 0.34	3.90± 2.13	0.98± 0.51	0.52± 0.21	4.46± 0.98
Friedmanniella	8.62± 2.31	7.00± 1.65	3.59± 1.45	17.7± 8.64	9.95± 3.42	2.52± 0.78	1.70± 0.45	7.00± 2.56	1.45± 0.45	1.61± 0.45	6.72± 2.05
Glaciecola	0.43± 0.19	0.89± 0.31	0.48± 0.15	1.22± 0.37	0.55± 0.23	1.23± 0.45	0.28± 0.13	0.15± 0.08	0.47± 0.19	0.61± 0.31	0.04± 0.02
Hymenobacter	0.82± 0.41	1.56± 0.45	0.88± 0.35	5.78± 2.63	5.91± 2.45	8.52± 2.31	0.11± 0.06	0.18± 0.05	0.35± 0.14	0.40± 0.19	0.26± 0.14

Loktanella	3.72± 1.25	1.84± 0.45	1.86± 0.67	1.16± 0.41	2.11± 0.25	3.05± 1.26	2.77± 1.21	3.13± 1.78	3.83± 1.32	2.84± 1.23	0.50± 0.23
Mycoplasma	0	0	0	0	0.07± 0.04	0	0	0	0	0	0
Octadecabacter	5.90± 1.34	4.23± 2.02	9.46± 3.45	3.08± 1.21	3.21± 1.06	4.94± 2.07	3.21± 0.98	1.85± 0.43	3.99± 0.45	2.46± 1.05	2.00± 1.21
Oleispira	0.62± 0.19	0.28± 0.11	0.47± 0.19	0.88± 0.29	1.12± 0.46	0.54± 0.17	1.11± 0.34	1.14± 0.65	0.84± 0.32	4.41± 0.87	0.14± 0.09
Phormidium	0.97± 0.31	2.09± 0.96	0.35± 0.15	6.17± 3.21	2.60± 1.31	1.87± 0.78	0.29± 0.15	4.54± 2.35	0.40± 0.09	0.43± 0.15	2.30± 0.86
Propionibacterium	0.71± 0.23	2.07± 1.02	0.20± 0.09	1.90± 0.41	0.37± 0.15	1.04± 0.41	3.99± 2.65	7.45± 2.66	4.49± 2.09	3.91± 1.08	14.7± 5.64
Pseudoalteromonas	0.44± 0.19	0.22± 0.10	1.81± 0.76	0.11± 0.05	0.19± 0.05	0.33± 0.12	0.72± 0.34	0.35± 0.19	0.36± 0.21	0.91± 0.32	1.68± 0.43
Proteus	0.03± 0.02	0.21± 0.05	0.01± 0.02	0.06± 0.03	0.004± 0.003	0.04± 0.02	0.06± 0.05	0.15± 0.10	0.07± 0.05	0.02± 0.04	0.06± 0.04
Sediminicola	0.32± 0.10	0.77± 0.23	0.12± 0.06	0.10± 0.04	0.09± 0.05	0.19± 0.07	0.02± 0.04	0.02± 0.03	0.07± 0.04	0.11± 0.06	0.0003± 0.0002
Sphingomonas	1.59± 0.46	2.56± 0.56	2.21± 0.54	4.31± 1.54	2.77± 1.31	3.01± 0.98	0.35± 0.15	1.20± 0.43	0.85± 0.39	0.66± 0.21	0.78± 0.24
Sulfurimonas	2.10± 1.02	0.48± 0.14	3.52± 1.23	0.89± 0.32	0.78± 0.25	1.71± 0.45	1.96± 0.76	0.93± 0.32	2.90± 0.86	0.14± 0.08	0.03± 0.05
Other	20.2± 10.1	23.3± 0.98	16.3± 10.4	12.1± 8.91	18.9± 10.1	25.1± 14.1	21.2± 7.87	22.4± 4.80	18.5± 5.67	13.2± 4.67	10.2± 4.56

Values correspond to the average and standard error for each of the taxa. Classes comprising <1% of the total number of sequences within a sample were simply classified as “Other”.
