# Supplementary material for: Distinct Temporal Succession of Bacterial Communities in Early Marine Biofilms in a Portuguese Atlantic Port
Source: Front Microbiol. 2020 Aug 11;11:1938. doi: 10.3389/fmicb.2020.01938 (PMC7432428; doi:10.3389/fmicb.2020.01938)
Supplement: TABLE S7 — SIMPER analysis showing the taxa most contributing to total dissimilarity of bacterial communities colonizing surfaces and the respective contribution to the dissimilarity in the different treatments. [file Table_7.docx]

Supplementary Table 7: SIMPER analysis showing the taxa most contributing to total dissimilarity of bacterial communities colonizing surfaces and the respective contribution to the dissimilarity in the different treatments.

| **Paint vs No Paint** | | **Biofilm vs Planktonic** | | **Spring vs Winter** | |
| --- | --- | --- | --- | --- | --- |
| **Taxon** | **Contribution %** | **Taxon** | **Contribution %** | **Taxon** | **Contribution %** |
| Halomonas | 4.86 | Halomonas | 4.43 | Mycoplasma | 3.43 |
| Mycoplasma | 3.61 | Cryomorphaceae | 3.72 | Friedmanniella | 3.02 |
| Methylococcales | 2.95 | Rhodobacteraceae | 3.61 | Stramenopiles | 2.74 |
| Polaribacter | 2.69 | Glaciecola | 3.31 | Clostridium | 1.96 |
| Flavobacteriaceae | 2.55 | Propionibacterium | 2.86 | Propionibacterium | 1.89 |
| Stramenopiles | 2.41 | Octadecabacter | 2.68 | Oleispira | 1.86 |
| Clostridium | 2.05 | Friedmanniella | 2.03 | Halomonas | 1.68 |
| Friedmanniella | 2.05 | Clostridium | 2.03 | Octadecabacter | 1.67 |
| Oleispira | 2.05 | Stramenopiles | 1.69 | Aurantimonadaceae | 1.66 |
| Alphaproteobacteria | 1.51 | Mycoplasma | 1.46 | Flavobacteriaceae | 1.65 |
| Lutibacterium | 1.29 | Flavobacteriaceae | 1.14 | Pseudoalteromonas | 1.60 |
| Octadecabacter | 1.21 | Pseudoalteromonas | 1.13 | Vibrionaceae | 1.07 |
| Pseudoalteromonas | 1.14 | Phormidium | 1.13 | Methylococcales | 1.02 |
| Methylococcales | 1.11 | Oleispira | 1.02 | Rhodobacteraceae | 0.98 |
| Gammaproteobacteria | 1.10 | Rhodobacteraceae | 0.96 | Glaciecola | 0.94 |
| Mycoplasma | 1.05 | Marinobacter | 1.90 | Loktanella | 0.92 |
| Marinobacter | 0.99 | Aurantimonadaceae | 0.95 | Cryomorphaceae | 0.86 |

Biofilms growing without and anti-corrosion paint (Paint *vs* No Paint); biofilms growing in all plates and planktonic communities (Biofilm vs planktonic), and season (Spring vs Winter).
